# An illustrated key to Neotropical species of the genus *Meteorus* Haliday (Hymenoptera, Braconidae, Euphorinae)

**DOI:** 10.3897/zookeys.489.9258

**Published:** 2015-03-23

**Authors:** Helmuth Aguirre, Luis Felipe de Almeida, Scott Richard Shaw, Carlos E. Sarmiento

**Affiliations:** 1 University of Wyoming, Department of Ecosystem Science and Management (3354), 1000 E. University Avenue, Laramie, WY 82071 USA; 2 Universidade Federal de São Carlos, Departamento de Ecologia e Biologia Evolutiva, Rod. Washington Luís, km 235, CEP 13565–905, São Carlos, SP, Brazil; 3 Laboratorio de Sistemática y Biología Comparada de Insectos, Instituto de Ciencias Naturales, Universidad Nacional de Colombia, A. A. 7495, Bogotá, Colombia

**Keywords:** Taxonomy, parasitoid, gregarious parasitism, solitary parasitism, Lepidoptera, host, distribution

## Abstract

A comprehensive key for 75 species of *Meteorus* distributed across 15 Neotropical countries is presented. Eleven new species from Bolivia, Costa Rica and Ecuador are described: *Meteorus albistigma*, *Meteorus carolae*, *Meteorus eurysaccavorus*, *Meteorus fallacavus*, *Meteorus flavistigma*, *Meteorus haimowitzi*, *Meteorus magnoculus*, *Meteorus martinezi*, *Meteorus microcavus*, *Meteorus noctuivorus* and *Meteorus orion*. Expanded range distributions are recorded for *Meteorus andreae*, *Meteorus farallonensis*, *Meteorus guineverae*, *Meteorus jerodi*, *Meteorus kraussi*, *Meteorus papiliovorus* and *Meteorus quimbayensis*. The host of *Meteorus jerodi* is reported for the first time: a noctuid larva feeding on Asteraceae. *Meteorus papiliovorus* is recorded attacking Papilionidae larvae in Ecuador, therefore displaying a similar host family preference as formerly documented from Costa Rica and Colombia.

## Introduction

The cosmopolitan genus *Meteorus* comprises at least 332 species worldwide with 70 species known in Central and South America (Yu 2012; Jones and Shaw 2012; Aguirre et al. 2014; Aguirre and Shaw 2014a, 2014b). The study of the Neotropical fauna has received particular attention in Colombia accounting for 38 species (Aguirre et al. 2011), Costa Rica with 21 (Zitani et al. 1998; Shaw and Nishida 2005; Barrantes et al. 2011) and Ecuador with 18 (Shaw and Jones 2009; Aguirre et al. 2010; Jones and Shaw 2012; Aguirre and Shaw 2014a, 2014b). In contrast, several other countries have far fewer species reported: Argentina with six species (Tosquinet 1900; Blanchard 1936; De Santis 1967; Luna and Sanchez 1999), Mexico with three (Marsh 1979; Pair et al. 1986; Molina-Ochoa et al. 2001), Brazil, Chile, Honduras, Nicaragua each with two (Porter 1926; Muesebeck 1939; Muesebeck 1958; Artigas 1972; Maes 1989; Gladstone 1991; Cave 1993), and Bermuda, Panama, Peru and Venezuela each with one (Ashmead 1889; Muesebeck 1939, 1967; Hilburn et al. 1990; De Huiza 1994). It seems likely that future exploration across the neotropics will yield many more new species of this genus.

*Meteorus* species develop as koinobiont endoparasitoids of Coleoptera and Lepidoptera larvae (Shaw and Huddleston 1991), but reports from Neotropical countries are restricted to 15 lepidopteran families (Yu 2012; Jones and Shaw 2012; Aguirre et al. 2014; Aguirre and Shaw 2014a, 2014b). There, the higher proportion of caterpillars parasitized by *Meteorus* belong to the family Erebidae (25%, 11 species) mainly in the subfamily Arctiinae (tiger moths), followed by Noctuidae and Pyralidae (14%, six species each one), Nymphalidae (11%, five species), and Megalopygidae (7%, three species).

*Zele* Curtis has been considered for long time as the sister-group to *Meteorus* within the tribe Meteorini, but a recent molecular phylogenetic analysis performed by Julia Stigenberg et al. (2015) for the subfamily Euphorinae concluded that *Zele* is embedded within *Meteorus*, hence rendering it a paraphyletic genus. Their conclusion agrees with an earlier analysis for the tribe Meteorini presented by Stigenberg and Ronquist (2011) and with the phylogenetic reconstruction published by Maeto (1990), although the internal relationships differ among these works. However, Stigenberg et al. (2015) remained cautious about any taxonomic status change until more comprehensive evidence can be evaluated. In this paper we treat species of *Meteorus sensu stricto* following Shaw’s (1997) definition of *Meteorus* exclusive of *Zele*: labrum completely concealed by clypeus; occipital carina present, complete or incomplete; epicnemial carina present; fore wing without vein 2cu-a, open first subdiscal cell; vein 3RSb straight; vein r-m present, forming a characteristic rhomboid or quadrate second submarginal cell; marginal cell of hind wing narrowed toward apex; vein m-cu absent; petiole at least 2.5 times wider at posterior margin than at narrowest point; metasomal terga with setae arranged in a single subapical row per tergum.

Huddleston (1980) discussed in depth the most relevant set of morphological characters employed in *Meteorus* taxonomy, which have been broadly used since then: relative size and shape of head related structures, the notauli distinctiveness, the presence of a pair of holes dorsally on the first tergite (dorsopes), the touching distance between the first tergite ventral borders, the ovipositor relative length and the shape of the tarsal claw are the most relevant. Huddleston pointed out upon the unreliable color variability in identifying species. In fact, color pattern is a variable that might be affected by environmental conditions (Abe et al. 2013) and may display a broad spectrum of change in species widely distributed. However, a careful examination of abundant species present in Colombia, Costa Rica and Ecuador support the use of such a trait in several cases.

In order to boost the *Meteorus* research in Neotropical countries this paper is intended to provide a compelling identification tool for those species described and recorded from Central and South America, in addition to describing 11 new species, and updating biological and geographical information for seven previously described species.

## Material and methods

Collections providing material are abbreviated below:

**UWIM** University of Wyoming Insect Museum, Laramie, Wyoming, USA;

**NMNH** Smithsonian National Museum of Natural History, Washington, USA;

**MACN** Museo Argentino de Ciencias Naturales Bernardino Rivadavia, Buenos Aires;

**ICN** Instituto de Ciencias Naturales, Universidad Nacional de Colombia, Bogotá.

Holotypes and paratypes of the new species are deposited at UWIM (See Suppl. material 1).

**Figure 1.**
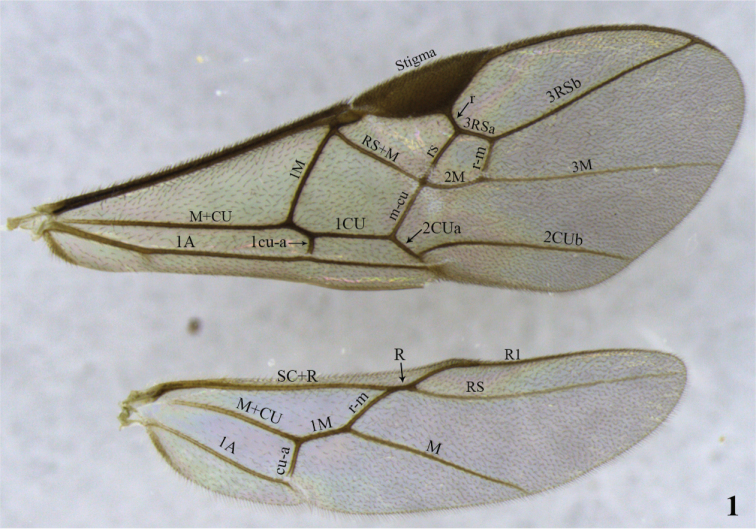
Wing venation nomenclature based on Sharkey and Wharton (1997).

**Figures 2–11.**
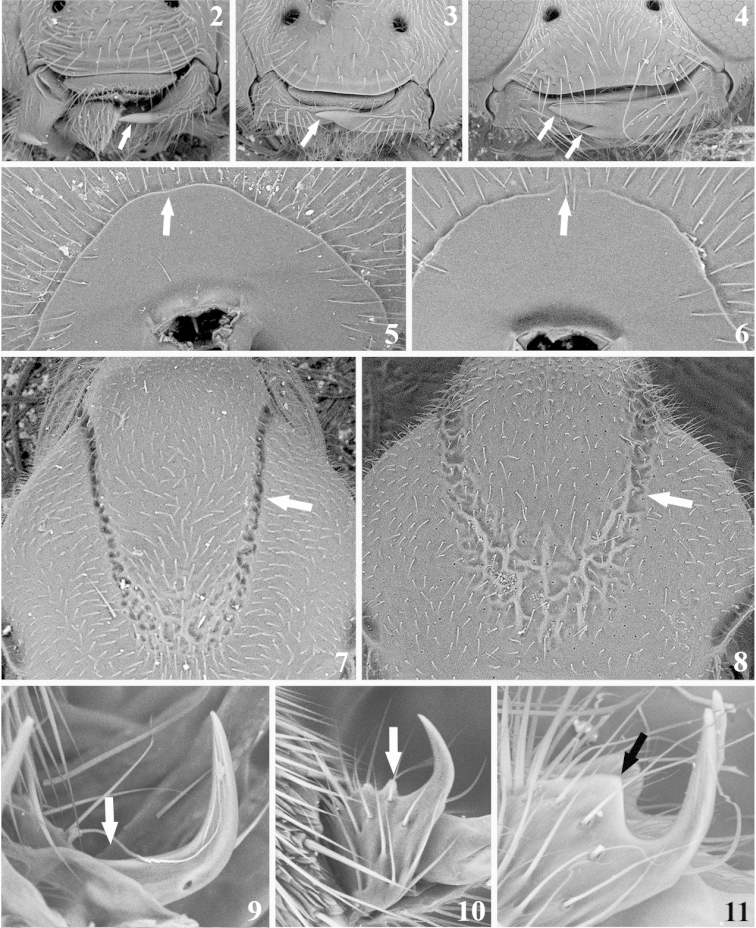
Morphological characters. Arrows on **2–4** indicate the mandible’s teeth: **2** twisted mandibles, look very thin in frontal view and only the upper teeth is visible **3** moderately twisted mandibles, look thicker in frontal view, sometimes the lower teeth is visible **4** mandibles not twisted, are the thickest in frontal view and both upper and lower teeth are visible **5** the arrow indicates the complete occipital carina **6** the arrow points the area where the occipital carina becomes incomplete **7–8** show mesoscutum in dorsal view; the arrows are pointing the notauli **7** notauli deep, distinct and linear **8** notauli shallow, obsolescent and indistinct **9–11** display three conditions present in tarsal claws **9** simple **10** with a small lobe **11** with a large lobe.

**Figures 12–19.**
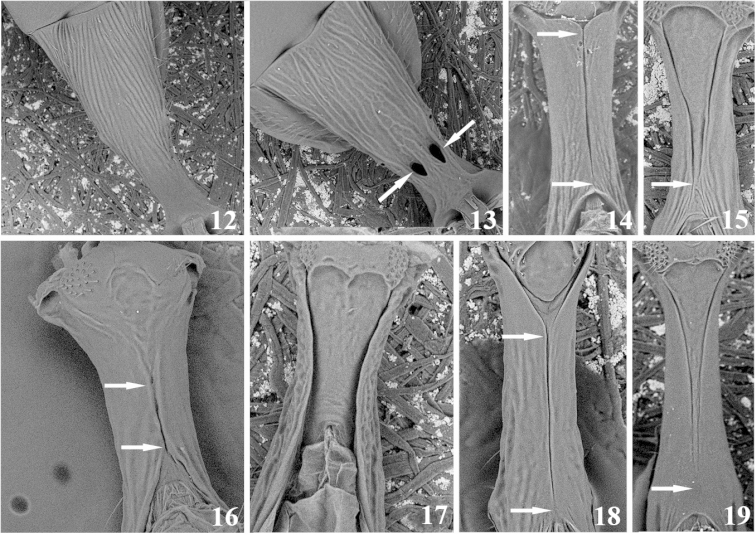
Morphological characters. **12–13** show the first metasomal tergite in dorsal view **12** first tergite without dorsopes **13** first tergite with a pair of dorsopes near the basal extreme (holes indicated by the arrows) **14–19** show the first metasomal tergite in ventral view; the portion’s structure pointing up is the anterior end. 14) Arrows indicate ventral borders of first tergite completely joined along ½ of segment **15** the arrow shows the distal extreme where the borders almost touch **16** arrows indicate the short section along which the ventral borders are touching **17** ventral borders widely separated **18** arrow on the top indicates the ventral borders basally separated, the arrow at the bottom shows them apically joined **19** the arrow signals the tergite’s apical portion where the ventral borders are either touching or fused.

**Figures 20–24.**
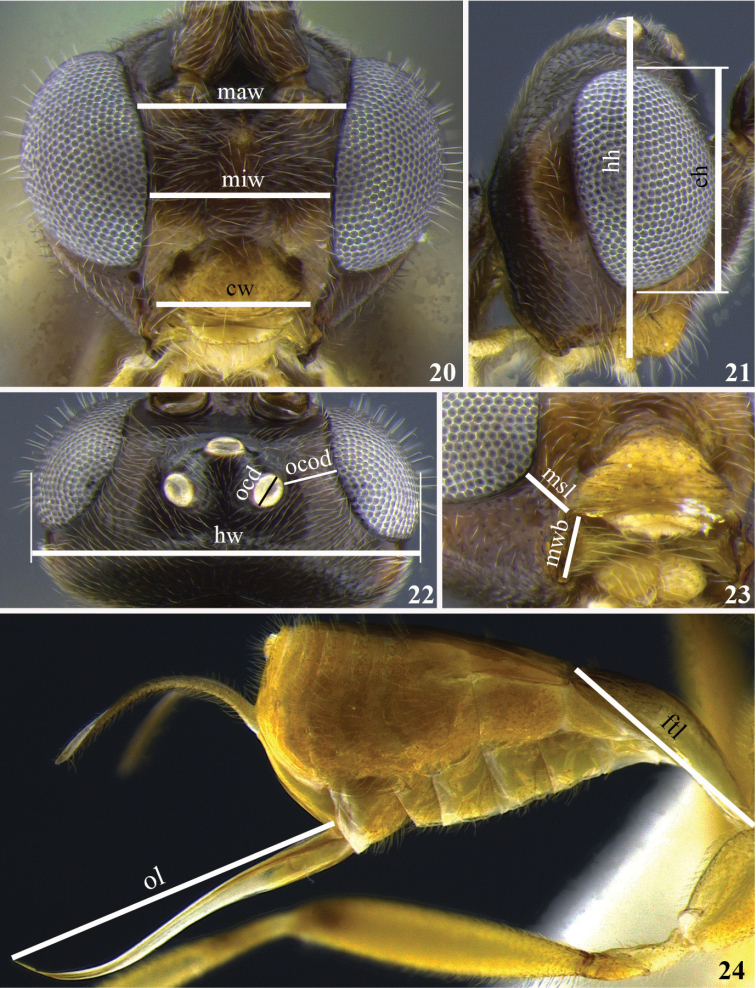
Morphometric characters. **20** Maw: face maximum width, miw: face minimum width, cw: clypeus width **21** hh: head height, eh: eye height **22** hw: head width, ocd: ocelar diameter, ocod: ocellus-ocular distance **23** msl: malar space length, mwb: basal mandible width **24** ftl: first tergite length, ol: ovipositor length.

General morphological terminology is based on Sharkey and Wharton (1997). The term precoxal sulcus is employed instead of sternaulus accordingly to Wharton (2006). Wing venation nomenclature employed in species descriptions is illustrated in Fig. 1. Sculpture related terms follow Harris (1979) and Aguirre et al. (2011). Specific terminology used in *Meteorus* taxonomy (based on Muesebeck 1923, Huddleston 1980, and Zitani et al. 1998) is represented in Figs 2–19. How to correctly position a specimen during morphometric examination is explained in Figs 20–24. In order to abbreviate descriptions, particularly explaining color details, metasomal tergites are sometimes referred as T1 (metasomal tergite number 1), T2 (metasomal tergite number 2) and so on. The specimens were measured using a Leica M80 stereomicroscope with micrometer on a 10× ocular. Images were captured with a Leica M205C stereomicroscope with digital Leica DFC295 camera kit and processed with Leica Application Suite Version 3.8.0 auto-montage software. Descriptions were made with the DELTA software (Dallwitz 1974, 1980). The software version for Windows 8 was downloaded from http://code.google.com/p/open-delta/.

Biological data of the new species described from Ecuador were collected as part of the project “Caterpillars and parasitoids in the Eastern Andes of Ecuador, CAPEA” (Dyer et al. 2014). Details about the field collecting process are described in Shaw and Jones (2009).

The key was built using morphological characters to distinguish all the species except in the couplet 60. *Meteorus eaclidis* and *Meteorus townsendi* present striking differences in cocoon construction and host use, being recorded on Saturniidae and Sphingidae caterpillars respectively. Such information support them as different species but are morphologically indistinguishable cryptic species.

The characters are based on examination of female specimens. Illustrations were embedded where either species differentiation may be challenging or the referred character(s) display some complexity.

## Results

### Key to the Neotropical species of *Meteorus*

**Table:** 

1	First metasomal tergite with dorsopes (as in Fig. 13)	**2**
–	First metasomal tergite without dorsopes (as in Fig. 12)	**14**
2	(1) Antennae with annuli; head and mesosoma mostly black; mandibles moderately twisted (as in Fig. 3); notauli deeply impressed and distinct (as in Fig. 7), tarsal claw with a small lobe (as in Fig. 10)	***Meteorus quimbayensis* Aguirre & Shaw**
–	Antennae without annuli; body color, mandibles, notauli and tarsal claw variable	**3**
3	(2) Surface of temples and genae coriaceous (Fig. 86); surface of second tergite coriaceous-costate (Fig. 90); front wing with vein 3RSb distinctly curved (Fig. 25); notauli shallowly impressed and not distinct (as in Fig. 8); occipital carina complete (as in Fig. 5); untwisted mandibles (as in Fig. 4); tarsal claw simple (as in Fig. 9); ventral borders of first tergite widely separated (as in Fig. 17)	***Meteorus eurysaccavorus* sp. n.**
–	Surface of temples, genae and second tergite of metasoma smooth; front wing with vein 3Rsb straight (as in Fig. 26); notauli deeply impressed and distinct (as in Fig. 7); occipital carina, mandibles, tarsal claw and ventral borders if the first tergite variable	**4**
	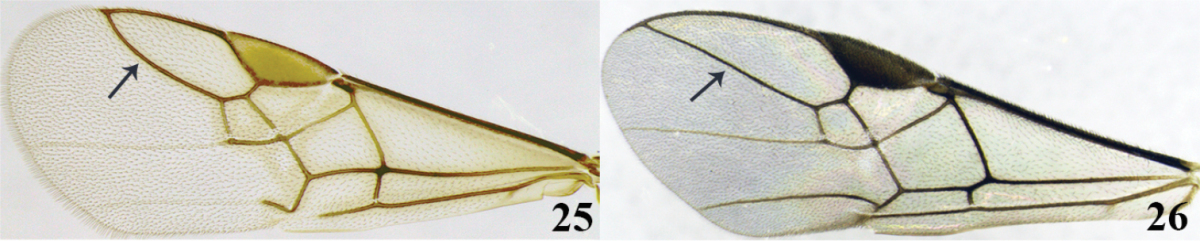	
4	(3) First tergite displaying only one color	**5**
–	First tergite with two colors, the one basally lighter than the one apically	**13**
5	(4) Carinae on propodeum present (as in Figure 27); ventral borders of first tergite widely separated (as in Figure 17)	**6**
–	Carinae on propodeum absent or obscured by complex sculpture (as in Figure 28); ventral borders of first tergite touching distally for a short distance (as in Figure 19)	***Meteorus fallacavus* sp. n.**
	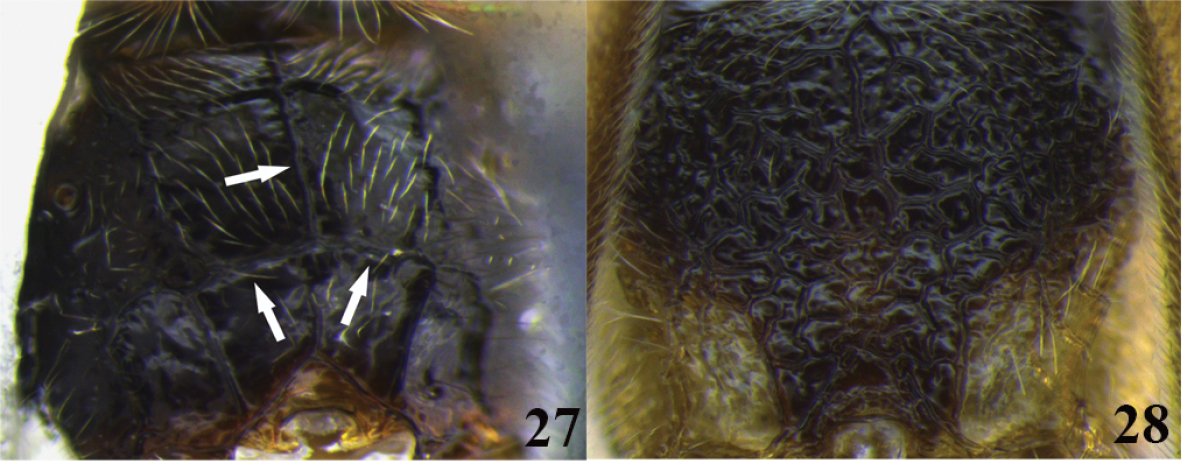	
6	(5) Untwisted mandibles (as in Figure 4)	**7**
–	Moderately twisted mandibles (as in Figure 3)	**10**
7	(6) Vertex in lateral view strongly convex and protruding above the ocelli (Fig. 29); occipital carina complete (as in Figure 5); tarsal claw simple (as in Figure 9)	***Meteorus magdalensis* Aguirre & Shaw**
–	Vertex in lateral view flattened (as in Figure 30), if slightly convex not protruding above the ocelli; occipital carina and tarsal claw variable	**8**
	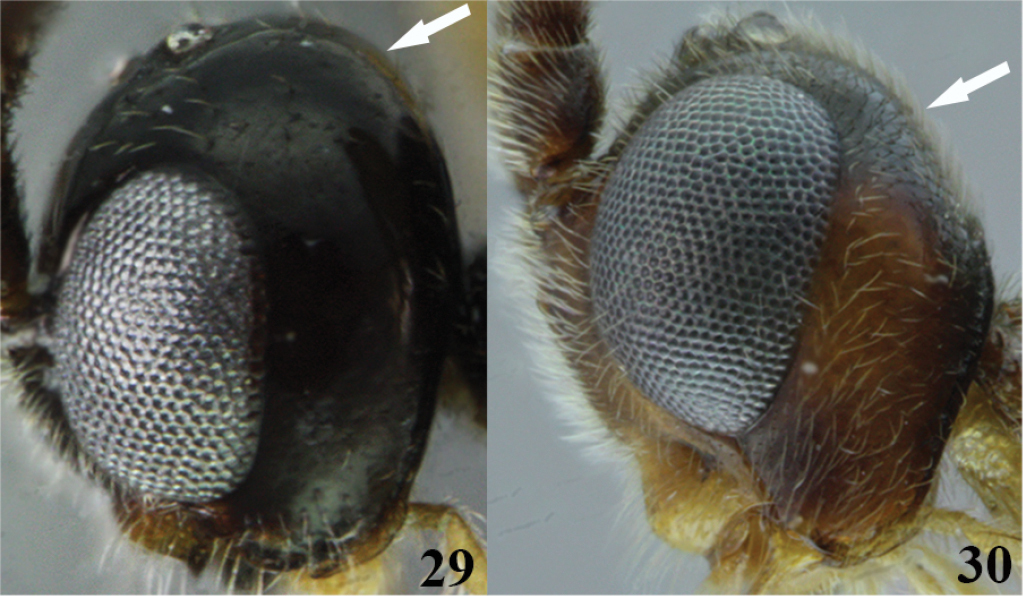	
8	(7) Occipital carina incomplete (as in Figure 6); eyes relatively large, head height/eye height = 1.4; eyes in frontal view convergent, maximum face width/minimum face width = 1.7; ocelli relatively large, ocellus-ocular distance/ocelar diameter = 1.7	***Meteorus santanderensis* Aguirre & Shaw**
–	Occipital carina complete (as in Figure 5); eyes relatively small, head height/eye height = 1.8–1.9; eyes in frontal view parallel, maximum face width/minimum face width = 0.9–1.1; ocelli relatively small, ocellus-ocular distance/ocelar diameter = 2.7–3.0	**9**
9	(8) Malar space short, malar space length 0.4 × mandible width basally (Figure 31); metapleuron smooth	***Meteorus guacharensis* Aguirre & Shaw**
–	Malar space longer, malar space length 0.9 × mandible width basally (Figure 32); metapleuron rugose	***Meteorus muiscai* Aguirre & Shaw**
10	(6) Tergites two and three mostly or totally yellow	**11**
–	Tergites two and three totally black-dark brown	**12**
	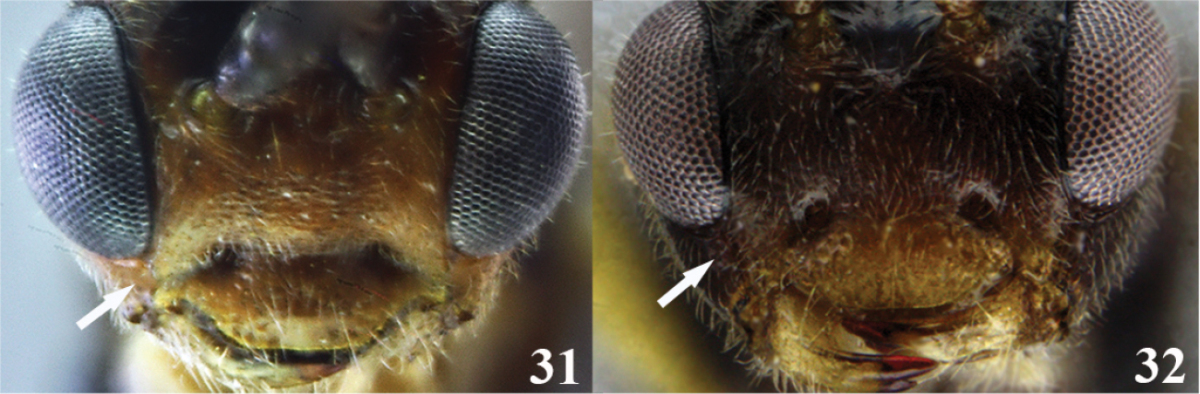	
11	(10) Mesopleuron totally black; antennae with 27–32 flagellomeres; occipital carina either complete or incomplete; tarsal claw either with a small lobe or simple (as in figures 9 and 10)	***Meteorus andreae* Aguirre & Shaw**
–	Mesopleuron mostly yellow; antennae with 22 flagellomeres; occipital carina complete (as in Figure 5); tarsal claw with a large lobe (as in Figure 11)	***Meteorus microcavus* sp. n.**
12	(10) Antennae with 34–35 flagellomeres; occipital carina incomplete (as in Fig. 6); tarsal claw with a large lobe (as in Fig. 11)	***Meteorus albisericus* Aguirre & Shaw**
–	Antennae with 26–32 flagellomeres; occipital carina complete (as in Fig. 5); tarsal claw either with a small or a large lobe (as in Figs 10 and 11)	***Meteorus guineverae* Aguirre & Shaw**
13	(4) Mesosoma and head mostly black; ocellus-ocular distance/ocelar diameter = 2.3–2.7; slightly convergent (Fig. 33), maximum face width/minimum face width = 1.1; mandibles untwisted (as in Fig. 4); tarsal claw simple (as in Fig. 9)	***Meteorus amazonensis* Aguirre & Shaw**
–	Mesosoma and head with black and testaceous patches; ocellus-ocular distance/ocelar diameter = 1.4; eyes in frontal view strongly convergent (Fig. 34), maximum face width/minimum face width = 1.7; mandibles moderately twisted (as in Fig. 3); tarsal claw with a small lobe (as in Fig. 10)	***Meteorus iguaquensis* Aguirre & Shaw**
	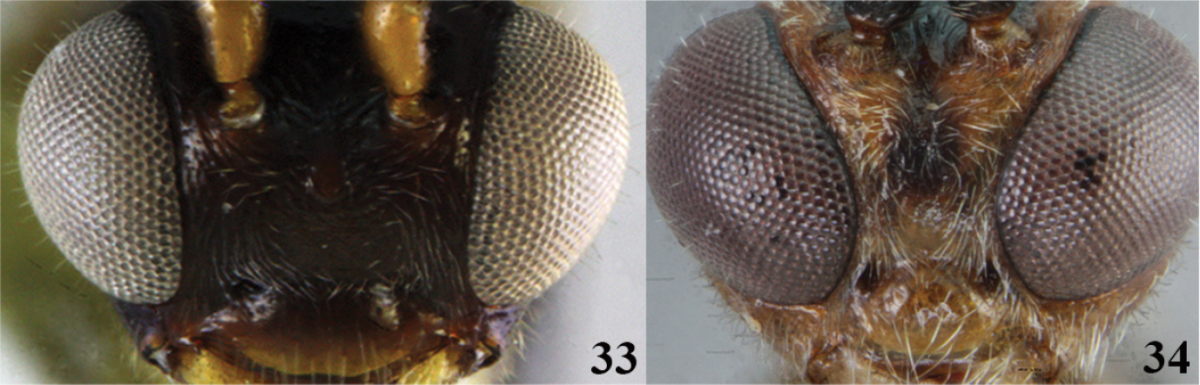	
14	(1) Precoxal sulcus absent, lateral surface of mesopleuron smooth (Fig. 35); occipital carina complete (as in Fig. 5); mandibles twisted (as in Fig. 2); notauli deeply impressed and distinct (as in Fig. 7); tarsal claw simple (as in Fig. 9); ventral borders of first tergite touching for a short distance (as in Fig. 16)	***Meteorus caritatis* Jones**
–	Precoxal sulcus present, lateral surface of mesopleuron with varied sculpture (as in Fig. 36); occipital carina, mandibles, notauli, tarsal claw and ventral borders of first tergite varible	**15**
	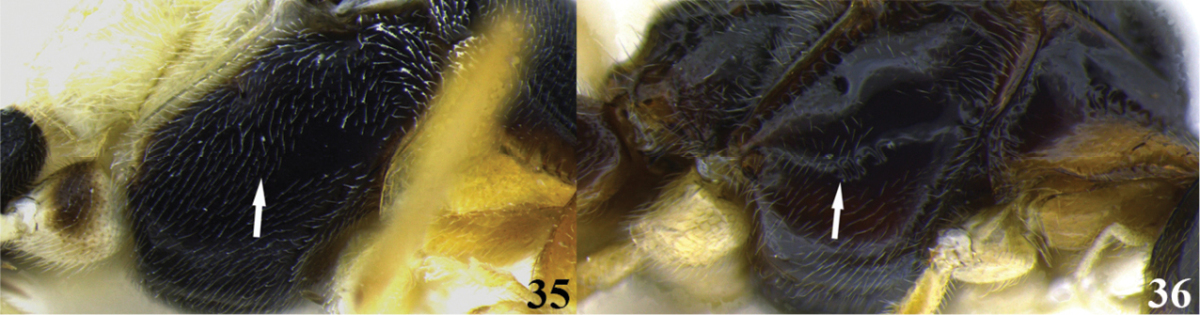	
15	(14) Presence of a pit on the frons (Fig. 37); body mostly yellow except some areas on mesonotum, mesopleuron ventrally, metanotum and propodeum brown; occipital carina complete (as in Fig. 5); notauli deeply impressed and distinctive (as in Fig. 7); tarsal claw simple (as in Fig. 9); ventral borders of the first tergite basally separated (as in Fig. 18)	***Meteorus bustamanteorum* Jones**
–	No pit on the frons (Fig. 38); body color, occipital carina, notauli, tarsal claw and ventral borders of the first tergite variable	**16**
	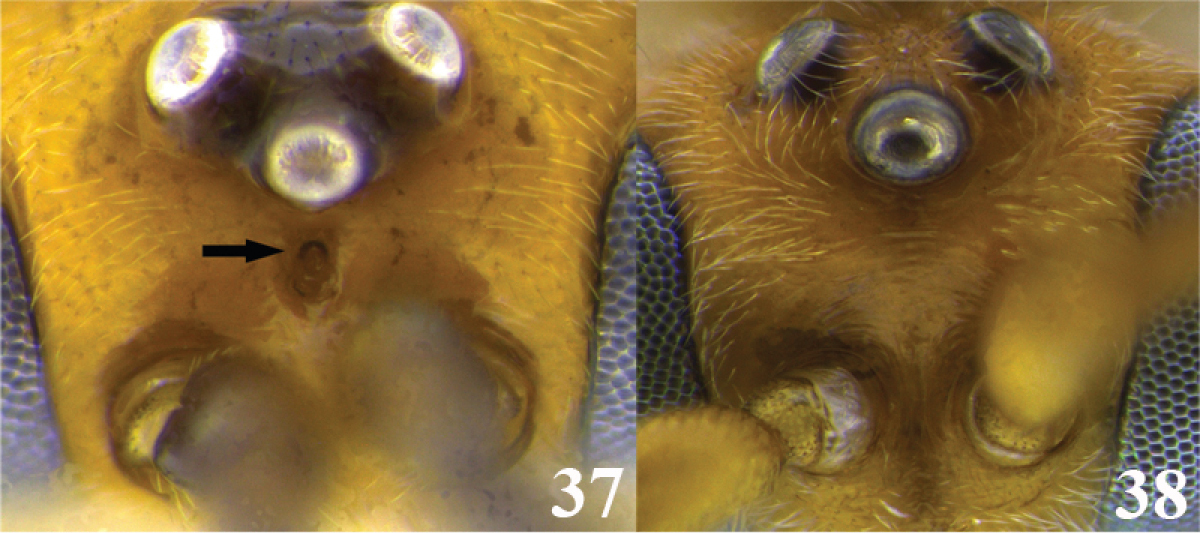	
16	(15) Pronotum and metapleuron coarsely rugose; scutellar disc strongly raised in a rounded point (Fig. 39); mandibles not twisted (as in Fig. 4); notauli deeply impressed and distinct (as in Fig. 7); tarsal claw simple (as in Fig. 9); ventral borders of first tergite completely joined along ½ of segment (as in Fig. 14)	***Meteorus corniculatus* Zitani**
–	Pronotum and metapleuron either smooth or sculptured but not as coarsely as before; scutellar disc convex (Fig. 40); mandibles, notauli, tarsal claw, and ventral borders of first tergite variable	**17**
	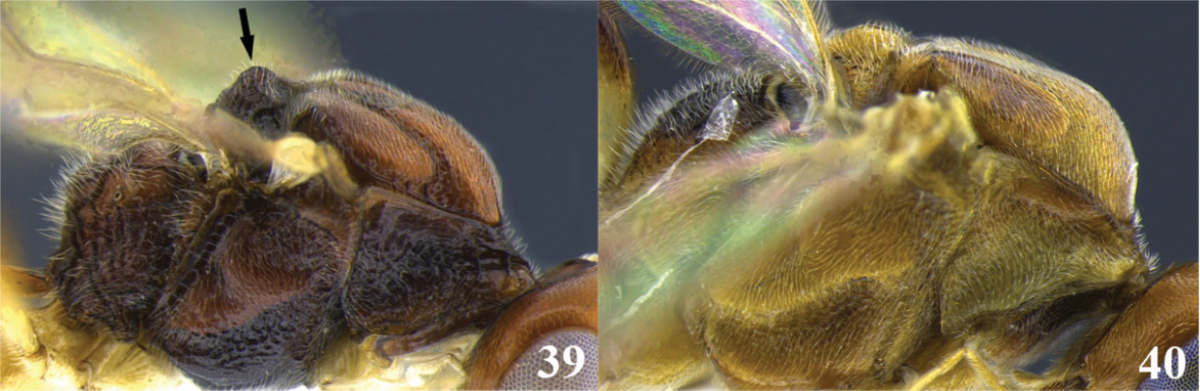	
17	(16) Front wing with vein r-m sinuated (Fig. 41); occipital carina complete (as in Fig. 5); mandibles moderately twisted (as in Fig. 3); notauli shallow and not distinct (as in Fig. 8); tarsal claw simple (as in Fig. 9); ventral borders of first tergite completely joined along ½ of segment (as in Fig. 14)	***Meteorus porcatus* Jones**
–	Front wing with vein r-m straight (as in Fig. 42); occipital carina, mandibles, notauli, tarsal claw and ventral borders of first tergite variable	**18**
	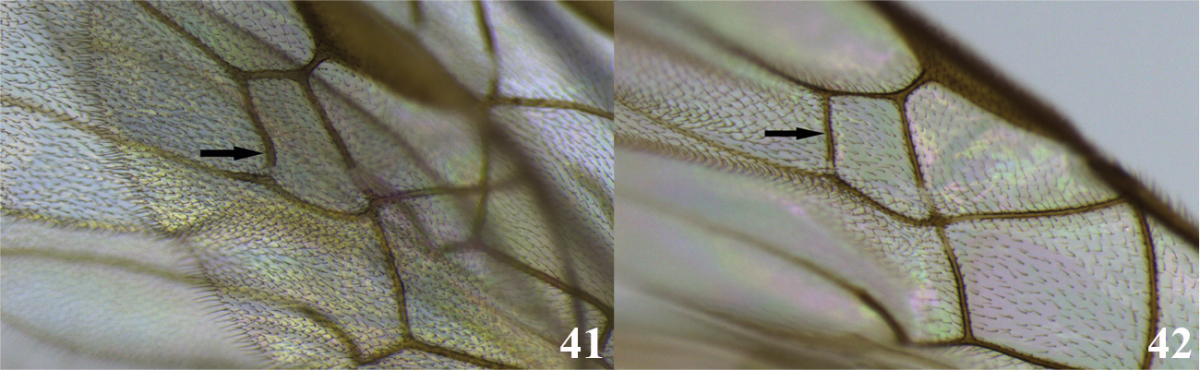	
18	(17) Clypeus coarsely sculptured and wrinkled (Fig. 43); occipital carina complete (as in Fig. 5); mandibles twisted (as in Fig. 2); notauli shallow and not distinct (as in Fig. 8); tarsal claw with a large lobe (as in Fig. 11); ventral borders of first tergite completely joined along ½ of segment (as in Fig. 14)	***Meteorus rugonasus* Shaw & Jones**
–	Clypeus with varied sculpture but not coarsely wrinkled (Fig. 44); occipital carina, mandibles, notauli, tarsal claw, and ventral borders of first tergite variable	**19**
	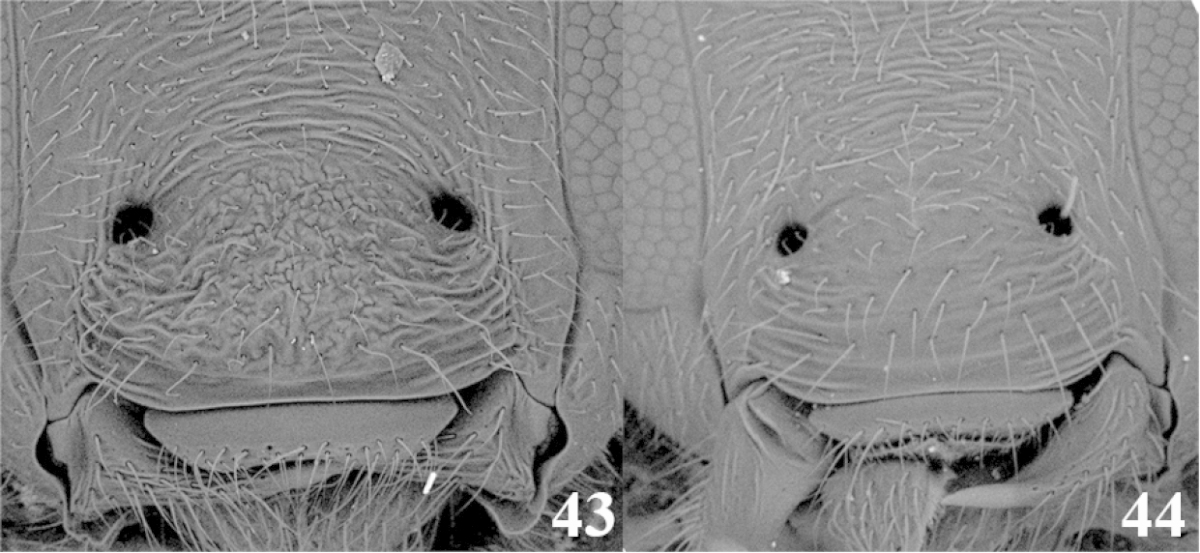	
19	(18) Mandibles completely twisted (as in Fig. 2)	**20**
–	Mandibles either moderately twisted or not twisted (as in Figs 3 and 4)	**61**
20	(19) Antennae with pale color at the tip (Fig. 45); occipital carina complete (as in Fig. 5); notauli shallow and not distinct (as in Fig. 8); tarsal claw with a large lobe (as in Fig. 11); ventral borders of first tergite completely joined along ½ of segment (as in Fig. 14)	***Meteorus rogerblancoi* Zitani**
–	Antennae dark to the tip (as in Fig. 46); occipital carina, notauli, tarsal claw and ventral borders if first tergite variable	**21**
	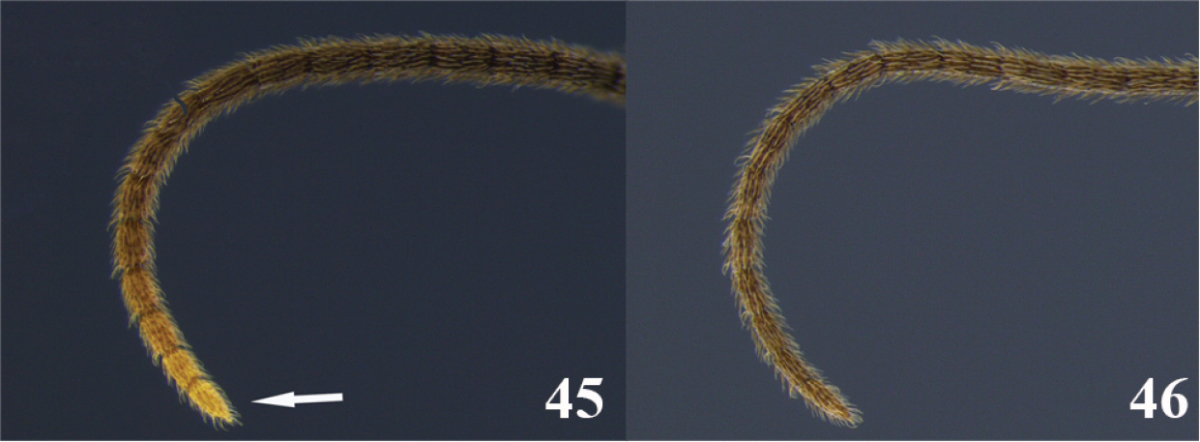	
21	(20) Occipital carina complete (as in Fig. 5)	**22**
–	Occipital carina incomplete (as in Fig. 6)	**45**
22	(21) Head completely yellow, orange or ferruginous except area among the ocelli black-dark brown; sometimes frons and vertex with brown patches but never occiput brown-black	**23**
–	Head color variable but occiput always brown-black	**35**
23	(22) Ventral borders of first tergite touching for a short distance (as in Fig. 16); notauli deeply impressed and distinct (as in Fig. 8); tarsal claw either simple or with a small lobe (as in Figs 9 and 10)	***Meteorus autographae* Muesebeck**
–	Ventral borders of first tergite completely joined along ½ of segment (as in Fig. 14); notauli shallow and not distinct (as in Fig. 8); tarsal claw variable	**24**
24	(23) Mesopleuron completely black-dark brown	**33**
–	Mesopleuron color variable, if it has either black or dark brown such colors cover just half or less of mesopleuron	**25**
25	(24) Abdominal tergites from 2 through 8 completely yellow, orange or ferruginous; tarsal claw variable	**26**
–	Abdominal tergites from 2 through 8 otherwise; tarsal claw with a large lobe (as in Fig. 11)	**29**
26	(25) Body mostly ferruginous; sometimes dark brown on propleuron, lateral mesonotal lobes, ventrally on mesopleuron, propodeum, and apically on first tergite; notauli shallow and not distinct (as in Fig. 8)	***Meteorus arizonensis* Muesebeck**
–	Body either mostly yellow or orange; notauli and tarsal claw variable	**27**
27	(26) Mesonotum orange but lateral mesonotal lobes black; eyes relatively small, head height/eye height = 1.6; ocelli relatively small, ocellus-ocular distance/ocelar diameter = 1.3; tarsal claw with a small lobe (as in Fig. 10)	***Meteorus luteus* Jones**
–	Mesonotum yellow; eyes relatively large, head height/eye height = 1.3–1.5; ocelli relatively large, ocellus-ocular distance/ocelar diameter = 0.8–1.2; tarsal claw with a large lobe (as in Fig. 11)	**28**
28	(27) Antennae with 29–34 flagellomeres	***Meteorus laphygmae* Haliday**
–	Antennae with 25 flagellomeres	***Meteorus euchromiae* Ashmead**
29	(25) Mesopleuron laterally yellow, ventrally black-dark brown	***Meteorus dos* Zitani**
–	Mesopleuron completely yellow	**30**
30	(29) Metanotum completely black-dark brown	***Meteorus imaginatus* Jones**
–	Metanotum dorsally brown-black, laterally yellow	**31**
31	(30) Hind coxa completely yellow; ocellus-ocular distance/ocelar diameter = 0.3; malar space length/mandible width basally = 0.1	***Meteorus haimowitzi* sp. n.**
–	Hind coxa basally yellow, apically brown; ocellus-ocular distance/ocelar diameter = 1.0–1.7; malar space length/mandible width basally = 0.7–0.9	**32**
32	(31) Ocellus-ocular distance/ocelar diameter = 1.2–1.7; head height/eye height = 1.5–1.6; gregarious cocoons set close to each other but suspended by individual threads	***Meteorus oviedoi* Shaw & Nishida**
–	Ocellus-ocular distance/ocelar diameter = 1.0; head height/eye height = 1.4; gregarious cocoons suspended together by a single cable	***Meteorus restionis* Shaw & Jones**
33	(24) Mesonotum and hind coxa completely dark brown; antennae with 25 flagellomeres; eyes convergent, face maximum width/minimum width = 1.5; tarsal claw with a small lobe (as in Fig. 10)	***Meteorus calimai* Aguirre & Shaw**
–	Mesonotum black-dark brown except area around notauli convergence point, as well as scutellum, yellow; hind coxa either partial or totally yellow; antennae with 30–33 flagellomeres; eyes parallel, face maximum width/minimum width = 1.1–1.2; tarsal claw with a large lobe (as in Fig. 11)	**34**
34	(33) Second tergite black-dark brown; hind coxa distally dark brown, basally yellow; tarsal claw with a particularly enlarged tarsal claw (as in Fig. 47)	***Meteorus zitaniae* Jones**
–	Second tergite dark brown with a yellow cup-shaped area along the middle; hind coxa completely yellow; tarsal claw with a large lobe but never as large as in *Meteorus zitaniae* (as in Fig. 48)	***Meteorus horologium* Jones**
	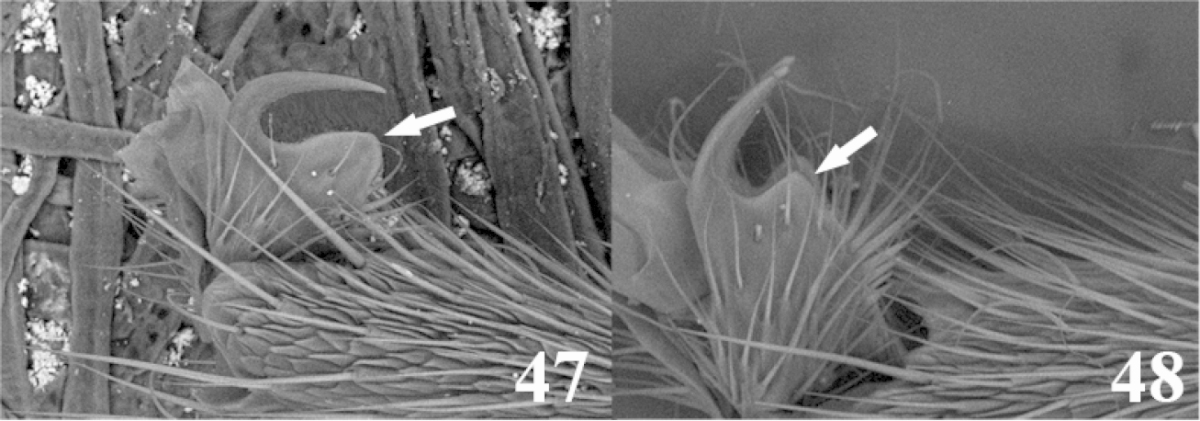	
35	(22) Ventral borders of first tergite either touching for a short distance (as in Fig. 16) or almost touching distally (as in Fig. 15)	***Meteorus pseudodimidiatus* Zitani**
–	Ventral borders of first tergite joined-fused along ½ of segment (as in Fig. 14) or separated basally (as in Fig. 18)	**36**
36	(35) Ventral borders of first tergite joined-fused along ½ of segment; notauli and tarsal claw variable	**38**
–	Ventral borders of first tergite separated basally; notauli deeply impressed and distinct (as in Fig. 7); tarsal claw with a large lobe (as in Fig. 11)	**37**
37	(36) Ovipositor curved (Fig. 49); first tergite basally yellow, distally brown; mesopleuron, metapleuron and propodeum mostly yellow	***Meteorus chingazensis* Aguirre & Shaw**
–	Ovipositor straight (Fig. 50); first tergite completely black; mesopleuron black and testaceous, metapleuron and propodeum black	***Meteorus dixi* Aguirre & Shaw**
	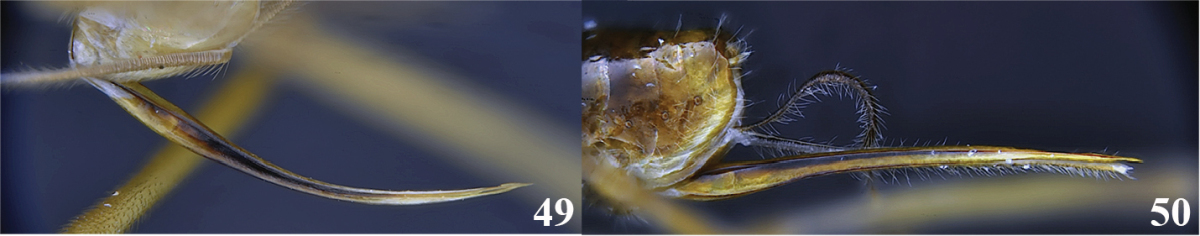	
38	(36) Mesosoma completely ferruginous; huge eyes, head height/eye height = 1.2–1.4; body large = 6.0–6.6 mm	***Meteorus magnoculus* sp. n.**
–	Mesosoma and eyes variable but not displaying the mentioned combination	**39**
39	(38) Tarsal claw simple (as in Fig. 9)	***Meteorus cecavorum* Aguirre & Shaw**
–	Tarsal claw with a large lobe (as in Fig. 11)	**40**
40	(39) Propodeum completely black-dark brown	**42**
–	Propodeum variable but not as before, if a black or dark brown area is present it is dorsally restricted	**41**
41	(40) Mesopleuron completely black; hind coxa dorsally black, ventrally white-yellow; head height/eye height = 1.5	***Meteorus pyralivorus* Aguirre & Shaw**
–	Mesopleuron orange except both dorsal and anterior borders black; hind coxa orange; head height/eye height = 1.3–1.4	***Meteorus desmiae* Zitani**
42	(40) First tergite completely black	***Meteorus anuae* Aguirre & Shaw**
–	First tergite basally white-yellow, distally brown-black	**43**
43	(42) Mesopleuron yellow	***Meteorus noctuivorus* sp. n.**
–	Mesopleuron brown-black	**44**
44	(43) Hind coxa dark brown; antennae with 24–27 flagellomeres; eyes convergent, face maximum width/minimum width = 1.4–1.6	***Meteorus carolae* sp. n.**
–	Hind coxa dorsally dark brown, ventrally yellow; antennae with 31 flagellomeres; eyes parallel, face maximum width/minimum width = 1.1	***Meteorus martinezi* sp. n.**
45	(21) Mesopleuron completely black-dark brown	**46**
–	Mesopleuron either yellow and black or yellow and dark brown	**51**
46	(45) Ventral borders of first tergite joined (eventually fused) along ½ of segment (as in Fig. 14); notauli variable	**47**
–	Ventral borders of first tergite touching for a short distance (as in Fig. 16), almost touching distally (as in Fig. 15) or separated basally (as in Fig. 18); notauli deeply impressed and distinct (as in Fig. 7)	**50**
47	(46) Body color with a notorious contrast of white-yellow on metapleuron and propodeum, dark brown on mesopleuron and hind coxa, and orange on mesonotum; notauli shallow and not distinct; tarsal claw with a small lobe	***Meteorus uno* Zitani**
–	Body color otherwise but not as before; if the general color pattern looks similar as the previous step, the mesonotum total or mostly black-dark brown	**48**
48	(47) Propodeum completely black; tarsal with a particularly enlarged tarsal claw (Fig. 47); notauli shallow and not distinct (as in Fig. 8)	***Meteorus zitaniae* Jones**
–	Propodeum otherwise but not as before; if any black or dark brown area present, it is in combination with either yellow or white areas; tarsal claw and notauli variable; if tarsal claw presents a large lobe, it is not as large as before (as in Fig. 48)	**49**
49	(48) Hind coxa completely dark brown; middle coxa completely yellow; notauli not distinct (as in Fig. 8)	***Meteorus orion* sp. n.**
–	Hind and middle coxa dorsally black, ventrally yellow; notauli distinct (as in Fig. 7)	***Meteorus mirandae* Aguirre & Shaw**
50	(46) Ventral borders of first tergite either touching for a short distance (as in Fig. 19) or almost touching distally (as in Fig. 15)	***Meteorus dimidiatus* (Cresson)**
–	Ventral borders of first tergite basally separated (as in Fig. 18)	***Meteorus oreoi* Jones**
51	(45) Notauli shallowly impressed and not distinct (as in Fig. 8); tarsal claw with a large lobe (as in Fig. 11)	**52**
–	Notauli deeply impressed and distinct (as in Fig. 7); tarsal claw variable	**57**
52	(51) Propodeum completely black	**53**
–	Propodeum otherwise but never completely black	**55**
53	(52) Mesonotal lobes black-dark brown; mesopleuron laterally yellow, ventrally dark brown	***Meteorus juliae* Aguirre & Shaw**
–	Mesonotal lobes and mesopleuron yellow	**54**
54	(53) Frons, vertex and temple black; wings slightly infuscated; head height/eye height =1.4–1.5; ovipositor length/ first tergite length = 1.7–1.8	***Meteorus margarita* Jones**
–	Frons, vertex and temple mostly orange-ferruginous; wings hyaline; head height/eye height = 1.6–1.7; ovipositor length/ first tergite length = 2.0–2.2	***Meteorus quasifabatus* Jones**
55	(52) Coxa orange and punctate; antennae with 30–35 flagellomeres; ocellus-ocular distance/ocelar diameter = 0.5–0.9; ovipositor length/ first tergite length = 2.3–3.2	**56**
–	Coxa basally yellow, apically brown, and strigate; antennae with 26–28 flagellomeres; ocellus-ocular distance/ocelar diameter = 1.0–1.4; ovipositor length/ first tergite length = 1.2–1.8	***Meteorus alejandromasisi* Zitani**
56	(55) Mesopleuron orange (body completely orange); vertex wide and slightly concave between lateral ocelli and occipital carina; antennae with 35 flagellomeres; ovipositor length/ first tergite length = 3.2	***Meteorus camilocamargoi* Zitani**
–	Mesopleuron orange-yellow medially, black dorso-anteriorly; vertex not as before; antennae with 30–31 flagellomeres; ovipositor length/ first tergite length = 2.3–2.6	***Meteorus desmiae* Zitani**
57	(51) Tarsal claw with a large lobe (as in Fig. 11); fore wing with second submarginal cell not narrowed anteriorly (Fig. 51); lateral borders of first tergite laterally flattened (Fig. 52)	***Meteorus sterictae* Zitani**
–	Tarsal claw simple (as in Fig. 9); fore wing with second submarginal cell narrowed anteriorly (as in Fig. 53); lateral borders of first tergite laterally convex (as in Fig. 54)	**58**
	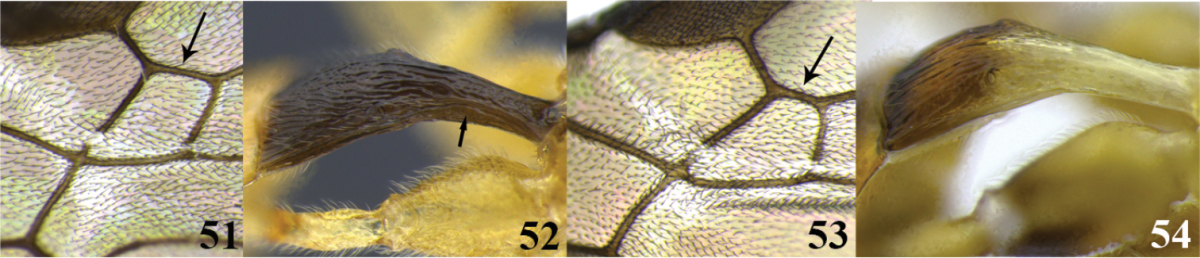	
58	(57) Mesonotum completely yellow-orange	**59**
–	Mesonotum with lateral lobes black-dark brown	***Meteorus papiliovorus* Zitani**
59	(58) Incomplete occipital carina (as in Fig. 6)	**60**
–	Complete occipital carina (as in Fig. 5)	***Meteorus congregatus* Muesebeck**
60	(59) Cocoons arranged in a compact mass encased in loose silk	***Meteorus townsendi* Muesebeck**
–	Cocoons arranged singly	***Meteorus eaclidis* Muesebeck**
61	(19) Mandibles moderately twisted (as in Fig. 3); notauli and tarsal claw variable	**62**
–	Mandibles not twisted (as in Fig. 2); notauli deeply impressed and distinct (as in Fig. 7); tarsal claw simple (as in Fig. 9)	**72**
62	(61) Ventral borders of first tergite joined completely along ½ of segment (as in Fig. 14)	**63**
–	Ventral borders of first tergite either touching for a short distance (as in Figs 16 and 19) or basally separated (as in Fig. 18)	**65**
63	(62) Mesopleuron completely black; notauli deeply impressed and distinct; tarsal claw with a large lobe	***Meteorus caquetensis* Aguirre & Shaw**
–	Mesopleuron otherwise; if any black area present on it, covering less than half of mesopleuron surface	**64**
64	(63) Propodeum completely yellow; notauli shallow and not distinct; tarsal claw simple; front wing with stigma brown	***Meteorus kraussi* Muesebeck**
–	Propodeum completely black; notauli deeply impressed; tarsal claw with a large lobe; front wing with stigma white	***Meteorus albumstigma* sp. n.**
65	(62) Ventral borders of first tergite touching for a short distance either medially (as in Fig. 16) or apically (as in Fig. 19)	**66**
–	Ventral borders of first tergite basally separated and joined along the rest of segment (as in Fig. 18)	**69**
66	(65) Notauli deeply impressed and distinct (as in Fig. 7); tarsal claw simple (as in Fig. 9)	**67**
–	Notauli shallow impressed and not distinct (as in Fig. 8); tarsal claw variable	**68**
67	(66) Small eyes (Fig. 55), head height/eye height = 1.8–1.9; ocellus-ocular distance/ocelar diameter = 2.6–3.2; eyes parallel in frontal view, face maximum width/minimum width = 1.1; ovipositor length/first tergite length = 1.3–1.8	***Meteorus micrommatus* Zitani**
–	Large eyes (Fig. 56); head height/eye height = 1.5; ocellus-ocular distance/ocelar diameter = 1.6; eyes convergent in frontal view, face maximum width/minimum width = 1.7; ovipositor length/first tergite length = 2.8	***Meteorus coffeatus* Zitani**
	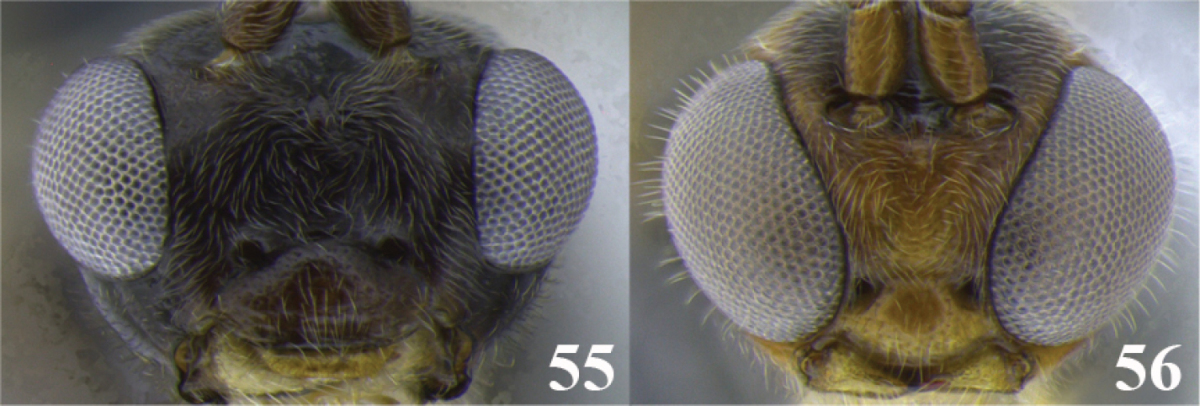	
68	(66) Eyes and ocelli large (Figs 57 and 58), head height/eye height = 1.2–1.4, ocellus-ocular distance/ocelar diameter = 0.6–0.7; occipital carina complete (as in Fig. 5)	***Meteorus antioquensis* Aguirre & Shaw**
–	Eyes and ocelli smaller (Figs 59 and 60), head height/eye height = 1.5–1.6, ocellus-ocular distance/ocelar diameter = 1.0–1.7; occipital carina incomplete (as in Fig. 6)	***Meteorus rubens* (Cresson)**
	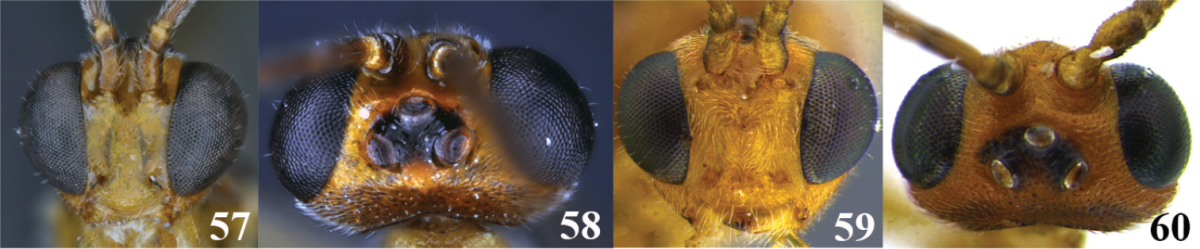	
69	(65) Tarsal claw with a large lobe (as in Fig. 11); occipital carina complete (as in Fig. 5); fore wing with yellow stigma	***Meteorus flavistigma* sp. n.**
–	Tarsal claw simple (as in Fig. 9); occipital carina variable; fore wing with stigma color variable	**70**
70	(69) Body completely or mostly yellow-orange; if it is mostly yellow-orange then metanotum, propodeum and tergites with brown areas; notauli variable; occipital carina incomplete (as in Fig. 6)	**71**
–	Body completely or mostly black-dark brown; notauli deeply impressed and distinct (as in Fig. 7); occipital carina complete (as in Fig. 5)	***Meteorus boyacensis* Aguirre & Shaw**
71	(70) Body completely yellow-orange; notauli shallow and not distinct (as in Fig. 8)	***Meteorus jerodi* Aguirre & Shaw**
–	Body mostly yellow-orange with metanotum, propodeum dorsally and metasomal tergites 1, 4–8 brown; notauli deeply impressed and distinct (as in Fig. 7)	***Meteorus chilensis* Porter**
72	(61) Head completely yellow-testaceous	***Meteorus huilensis* Aguirre & Shaw**
–	Head either completely black-dark brown or black-dark brown except face testaceous	**73**
73	(72) Ventral borders of first tergite widely basally separated, distally either touching for a short distance (as in Fig. 19) or almost touching (as in Fig. 15); notauli posteriorly oval-shaped (Fig. 61)	**74**
–	Ventral borders of first tergite basally separated and joined along almost ½ of segment (as in Fig. 18); notauli converging posteriorly in a distinct v-shape (as in Fig. 62)	***Meteorus mariamartae* Zitani**
	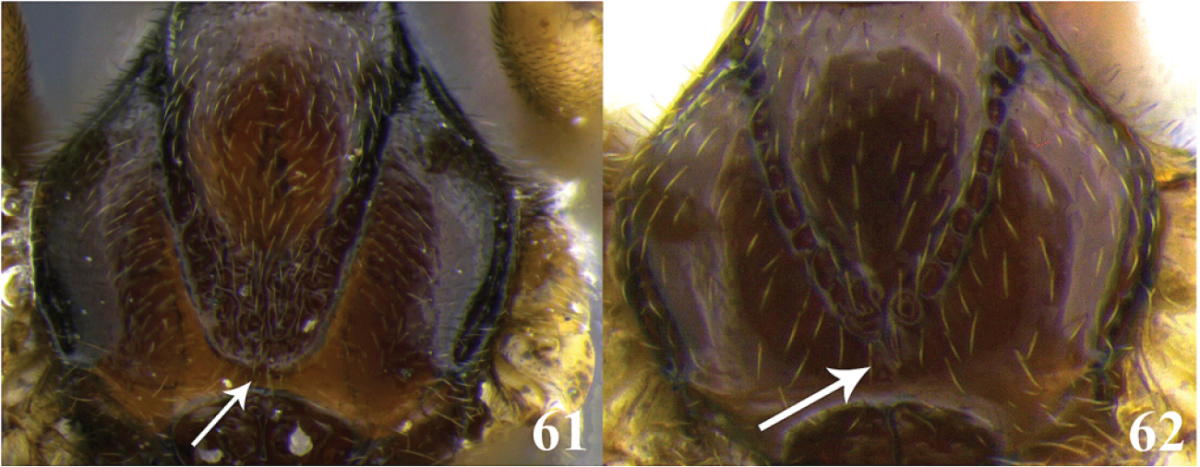	
74	(73) Eyes protuberant (Fig. 63); body usually large, body length = 4.0–9.7 mm	**75**
–	Eyes not protuberant (Fig. 64); body always small, body length = 2.5–3.7 mm	***Meteorus yamijuanum* Zitani**
	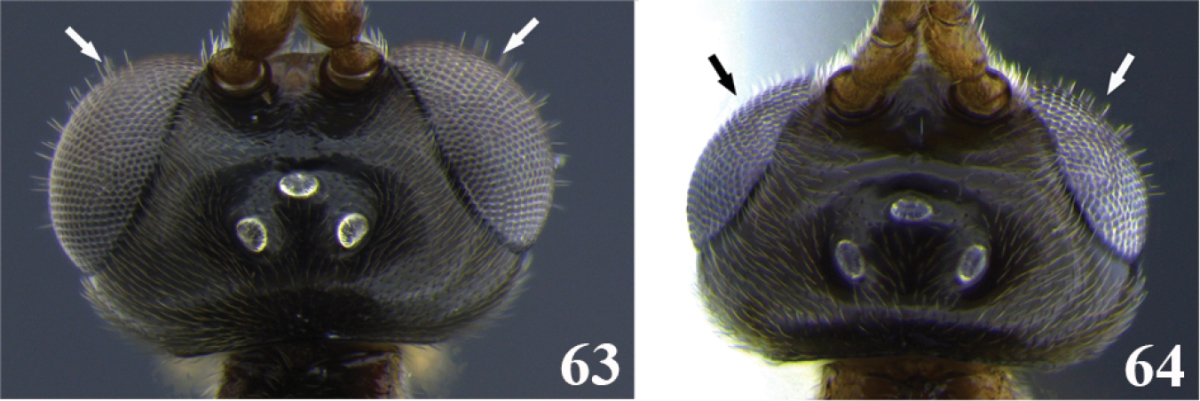	
75	(74) Antennae with 30–34 flagellomeres; body length = 8–9.7 mm; fore and middle coxa black; face maximum width/minimum width = 1.3–1.4	***Meteorus gigas* Aguirre, Shaw & Jones**
–	Antennae with 20–25 flagellomeres; body length = 4.7–5.9 mm; fore and middle coxa yellow; face maximum width/minimum width = 1.5–1.9	***Meteorus megalops* Zitani**

### Species not included in the key

***Meteorus australis* Tosquinet, 1900.**

Known only from Argentina. Type missed.

***Meteorus deltae* Blanchard, 1936.**

Known only from Argentina. Type missed.

***Meteorus eumenidis* Brethes, 1903.**

Zitani (2003) reported the transferring of *Meteorus eumenidis* Brethes, 1903 to the genus *Homolobus* Forster, 1862 after the examination by Michael Sharkey of the holotype deposited in the Museo Argentino de Ciencias Naturales. The *Meteorus eumenidis* holotype has the first metasomal tergite sessile, not petiolate, the first subdiscal cell of the fore wing closed, and the fore wing vein 3RSb curved towards the posterior wing margin (Zitani 2003).

***Meteorus laqueatus* Enderlein, 1920.**

The holotype of *Meteorus laqueatus* deposited at the Zoological Museum in Warsaw, Poland, was examined by Nina Zitani (Zitani 2003), who concluded that, based on the broadening of the marginal cell of the hind wing and the scattered setae on the metasomal tergites, this species should be assigned to the genus *Zele* Curtis, 1832.

***Meteorus platensis* Brethes, 1913.**

**Figures 65–66.**
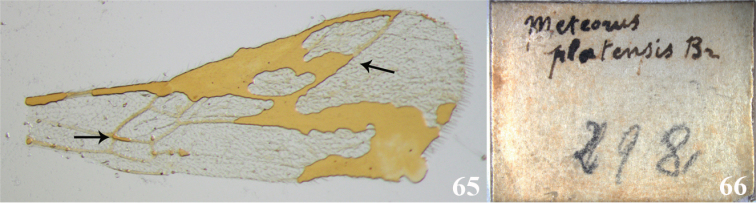
*Meteorus platensis*. **65** Front wing; the arrow on the left shows a small-rhomboid first discal cell, the arrow on the right indicates the short and curved 3RSb vein **66** type label.

Juan Jose Martinez from the Museo Argentino de Ciencias Naturales examined and provided an image of the *Meteorus platensis* holotype (Figs 65–66). Just the forewing remains and it is in very bad condition but the small and rhomboid first discal cell (arrow on the left Fig. 65), and the short and slightly curved vein 3RSb (arrow on the right Fig. 65) are clear enough to conclude it is not *Meteorus*. The visible pattern of venation is more consistent with it possibly belonging to the Opiinae or Alysiinae.

### Description of new species

#### Meteorus albistigma

Taxon classificationAnimaliaHymenopteraBraconidae

Aguirre, Almeida & Shaw sp. n.

http://zoobank.org/F1302EC9-38DA-4B46-9952-D02D701026C6

Figures 67–72

##### Diagnosis.

Occipital carina complete; eyes convergent, face maximum width 1.8 × minimum width; mandibles moderately twisted; notauli deeply impressed, distinctive and foveolate; propodeum aerolate-rugose and absent of both carinae and a median depression; hind coxa punctuate-polished; tarsal claw with large lobe; dorsopes absent; ovipositor 2.7 × longer than first tergite, stigma white.

**Figures 67–72.**
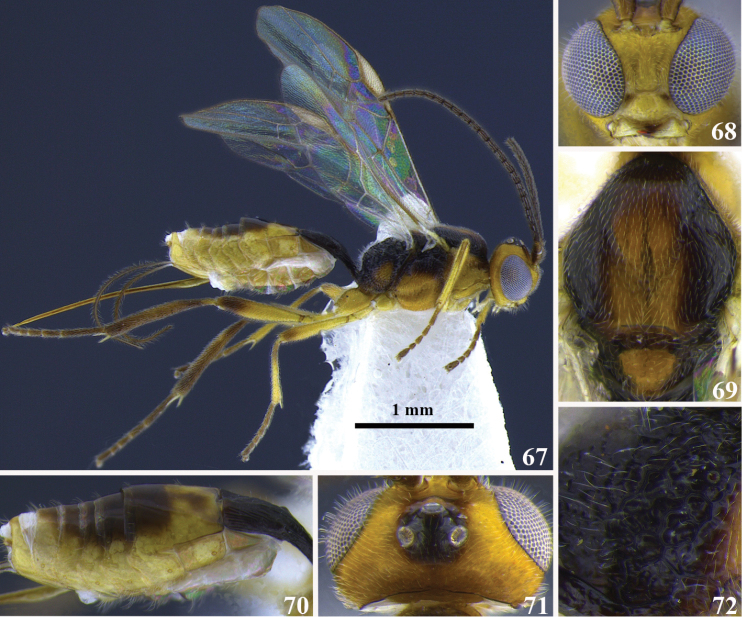
*Meteorus albistigma* sp. n. **67**) Female in lateral habitus **68** head in frontal view **69** mesoscutum in dorsal view **70** metasoma in dorso-lateral view **71** head in dorsal view **72** propodeum.

##### Body color.

Antenna dark brown, annulus absent; head yellow except area between ocelli black. Propleuron and pronotum yellow; mesonotum black except yellow among mesonotal lobes and on the scutellum; mesopleuron orange except black close to the tegula; metanotum totally black; metapleuron orange; propodeum black. Prothoracic legs yellow except tarsus light brown; mesothoracic legs yellow except femur apically, tibia and tarsus brown; metathoracic legs yellow except tibia brown, femur apically and tarsus dark brown. T1 black, T2 yellow, T3 brown, T4–T6 brown medially and yellow laterally, T7–T8 yellow; sterna yellow. Wing membrane hyaline; stigma white.

##### Body length.

3.1 mm.

##### Head.

Antenna with 20 flagellomeres (antenna broken); flagellar length/width ratios as follows: F1 = 4.4, F2 = 4, F3 = 3, F18 = 1.3, F19 = 1.3, F20 = 2.2; head 1.1 wider than high; occipital carina incomplete; ocellus-ocullar distance 1.5 × ocellar diameter; head height 1.6 × eye height; temple length 0.4 × eye length in dorsal view; vertex in dorsal view not descending vertically behind the lateral ocelli; frons smooth and polished; face maximum width 1.8 × minimum width; face surface irregular and shiny; face minimum width 0.7 × clypeus width; clypeus surface irregular and shiny; malar space length 0.4 × mandible width basally; mandibles moderately twisted.

##### Mesosoma.

Pronotum in lateral view carinate; propleuron smooth; notauli deeply impressed, distinctive and foveolate; mesonotal lobes well defined; central lobe of mesoscutum either punctuate or smooth and polished; scutellar furrow with three carinae; mesopleuron punctate; precoxal sulcus short, narrow and foveate-lacunose; metapleuron mostly smooth but rugose close to the hind coxa; propodeum aerolate-rugose and absent of both carinae and a median depression.

##### Legs.

Hind coxa punctuate-polished; tarsal claw with large lobe.

##### Wings.

Wing length 2 mm. Front wing: second submarginal cell not strongly narrowed anteriorly; length of vein r 0.6 × length of vein 3RSa; vein 3RSb straight; length of vein 3RSa equal to length of vein r-m; vein m-cu antefurcal. Hind wing: length of vein 1M equal to length of vein cu-a; length of vein 1M 0.9 × length of vein r-m.

##### Metasoma.

Dorsopes absent; ventral borders of first tergite joined completely along ½ of segment; first tergite rugulose-costate, the costae convergent; ovipositor thickened basally and straight; ovipositor 2.7 × longer than first tergite; T2–T7 smooth.

##### Cocoon.

Unknown.

##### Female variation.

Unknown.

##### Male variation.

Unknown.

##### Type locality.

COSTA RICA, Alajuela, Chiles de Aquas, Zarcas Cafe, 300 m.

##### Type specimen.

Holotype female (point mounted). Original label: COSTA RICA, Alajuela, Chiles de Aquas, Zarcas Cafe, 300 m, collected XI.1989, R. Cespedes leg., UWIM.

##### Distribution.

Costa Rica, at the province of Alajuela.

##### Biology.

Unknown.

##### Comments.

*Meteorus albistigma* resembles *Meteorus kraussi* in having the ventral borders of first tergite completely fused along ½ of segment and mandibles moderately twisted. *Meteorus albistigma* can be separated by having the propodeum dorsally dark (completely or mostly yellow in *Meteorus kraussi*), the notauli deeply impressed (shallow and not distinct in *Meteorus kraussi*), the tarsal claw with a large lobe (tarsal claw simple in *Meteorus kraussi*) and the stigma of the front wing white (brown in *Meteorus kraussi*).

##### Etymology.

The name of this species is composed by the latin prefix “albi”, meaning white, and the stem “stigma” because of the color of this structure on the front wing.

#### Meteorus carolae

Taxon classificationAnimaliaHymenopteraBraconidae

Aguirre, Almeida & Shaw sp. n.

http://zoobank.org/2F7F4D96-6BD6-4E63-990C-6761AABB5DB0

Figures 73–79 , 80–82

##### Diagnosis.

Occipital carina complete; face maximum width 1.5 × minimum width; mandibles twisted; notauli shallow, not distinctive and rugose; propodeum aerolate-rugose; hind coxa strigate; tarsal claw with large lobe; dorsope absent; ventral borders of first tergite joined completely along ½ of segment; ovipositor 2.9 × longer than first tergite; body mostly dark brown.

**Figures 73–79.**
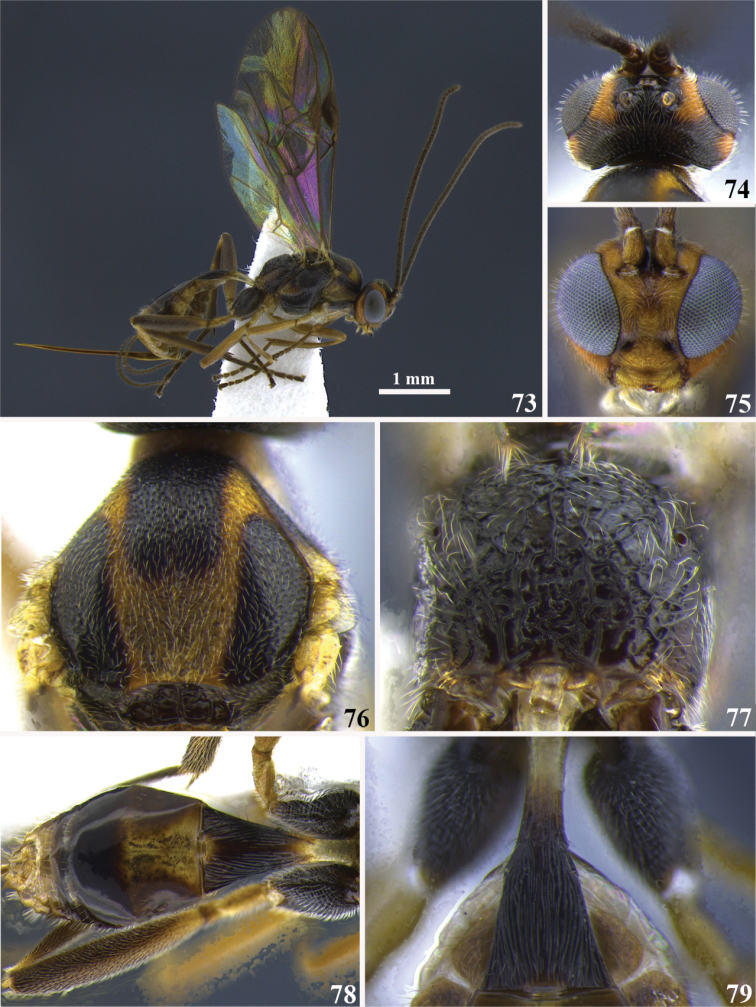
*Meteorus carolae* sp. n. female. **73** Habitus in lateral view **74** head in dorsal view **75** head in frontal view **76** mesoscutum in dorsal view **77** propodeum in posterior view **78** metasoma in dorsal view **79** First tergite in dorsal view.

**Figures 80–82.**
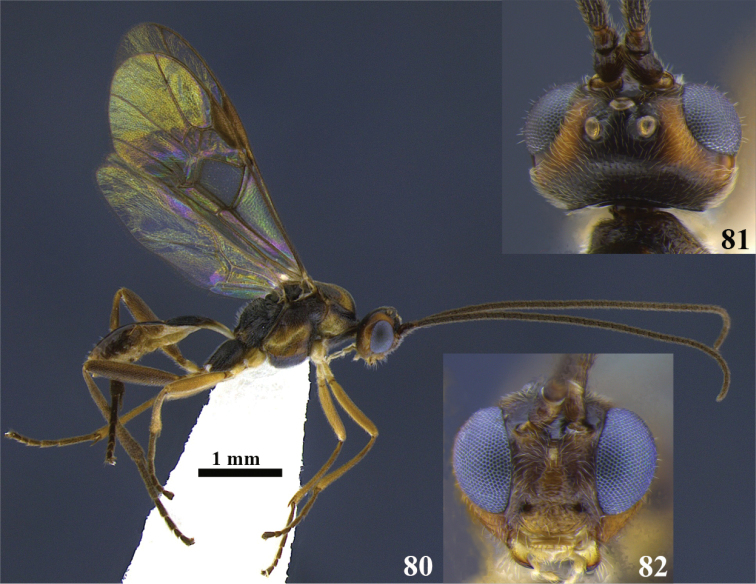
*Meteorus carolae* sp. n. male. **80** Habitus in lateral view **81** head in dorsal view **82** head in frontal view.

##### Body color.

Antenna dark brown; annulus absent; face and clypeus yellow; frons black on the middle and orange laterally; vertex orange between the lateral ocelli and the compound eyes; area around and among ocelli, vertex behind the lateral ocelli, temple and the most of the gena black; a small orange area of the gena along the compound eye. Propleuron dark brown; pronotum dorsally dark brown, then gradually becomes light brown to orange ventrally; mesonotal lobes black; area among lobes, notauli and scutellum yellow-orange; mesopleuron, metanotum, metapleuron and propodeum black. Prothoracic legs yellow; mesothoracic legs yellow except tarsus brown; metathoracic coxa dark brown, remaining leg light brown. T1 yellow basally, dark brown apically; T2 yellow basally, remaining brown; sterna yellow-cream. Wings hyaline; stigma dark brown.

##### Body length.

3.6 mm.

##### Head.

Antenna with 26 flagellomeres; flagellar length/width ratios as follows: F1 = 4, F2 = 3.7, F3 = 2.7, F24 = 1.5, F25 = 1.3, F26 = 1.8; head 1.2 wider than high; occipital carina complete; ocellus-ocullar distance 1.2 × ocellar diameter; head height 1.4 × eye height; temple length 0.4 × eye length in dorsal view; vertex in dorsal view not descending vertically behind the lateral ocelli; frons strigulate; face maximum width 1.5 × minimum width; face strigate-rugulose; face minimum width 0.8 × clypeus width; clypeus rugulose; malar space length 0.3 × mandible width basally; mandibles twisted.

##### Mesosoma.

Pronotum in lateral view extensively rugose; propleuron slightly puncticulate; notauli shallow, not distinctive and rugose; mesonotal lobes not well defined; central lobe of mesoscutum punctate; scutellar furrow with five carinae; mesopleuron punctate, rugose-lacunose close to the tegula; precoxal sulcus long, wide and rugose; metapleuron rugose; propodeum aerolate-rugose, both carinae or median depression absent.

##### Legs.

Hind coxa strigate; tarsal claw with large lobe.

##### Wings.

Wing length 3 mm; second submarginal cell of forewing not strongly narrowed anteriorly. Front wing: length of vein r 0.8 × length of vein 3RSa; vein 3RSb straight; length of vein 3RSa 0.8 × length of vein r-m; vein m-cu antefurcal. Hind wing: length of vein 1M 1.3 × length of vein cu-a; length of vein 1M equal to length of vein r-m.

##### Metasoma.

Dorsope absent; ventral borders of first tergite joined completely along ½ of segment; first tergite with costae convergent posteriorly; ovipositor thickened basally and straight; ovipositor 2.9 × longer than first tergite.

##### Cocoon.

Unknown.

##### Female variation.

Head face and clypeus light brown-honey; frons medially black, laterally orange; area between ocelli, temples and vertex behind the lateral ocelli black; gena orange. Pronotum dorsal border black, remaining yellow; median mesonotal lobe and scutellum light brown, lateral mesonotal lobes dark brown, area among lobes and notauli yellow; mesopleuron black except a medial-posterior patch yellow; metanotum totally black; metapleuron yellow, or orange except ventral border black; propodeum black; mesothoracic legs coxa, trochanter and trochantellus white, remaining dark brown; body length 3.2–3.7 mm; antenna with 24–27 flagellomeres; ocellus-ocullar distance 1–1.5 × ocellar diameter; temple length 0.5–0.6 × eye length in dorsal view; face maximum width 1.4–1.6 × minimum width; clypeus punctate; propleuron rugulose; precoxal sulcus short and wide; wing length 3.5 mm. Front wing: length of vein 3RSa 1–1.2 × length of vein r-m. Vein m-cu of forewing either intersticial or postfurcal. Ovipositor 2.3 × longer than first tergite.

##### Male variation.

Lateral lobes of mesonotum and apical area of median one black, yellow the rest; mesopleuron either yellow except area close to the tegula dark brown, or orange on the middle, black dorsally and ventrally; prothoracic and mesothoracic legs yellow except tarsus brown; metathoracic legs yellow except tibia brown, femur apically and tarsus dark brown; T2 yellow-orange basally, remaining dark brown; body length 3.8 mm; antenna with 32 flagellomeres; head height 1.1 × eye height; ocellus-ocullar distance 1.1 × ocellar diameter; head height 1.5 × eye height; face maximum width 1.2 × minimum width; face minimum width 0.9 × clypeus width; malar space length 0.5 × mandible width basally; wing length 3.4 mm. Front wing: length of vein r 0.6 × length of vein 3RSa. Hind wing: length of vein 1M equal to length of vein cu-a; length of vein 1M 0.8 × length of vein r-m. First tergite costate-reticulate.

##### Type locality.

COSTA RICA, Cartago, Dulce Nombre, Vivero Linda Vista, 1400 m.

##### Type specimen.

Holotype female (point mounted). Original label: COSTA RICA, Cartago, Dulce Nombre, Vivero Linda Vista, 1400 m, collected VI–VIII.1993, UWIM.

Paratypes. One female (point mounted), COSTA RICA, Cartago, 4km NE Cañón Génesis II, 2350 m, collected IV–V.1996, P. Hanson leg., UWIM. One female (point mounted), COSTA RICA, Cartago, 4 km NE Cañón Génesis II, 2350 m, collected V.1995, P. Hanson leg., UWIM. One male (point mounted), COSTA RICA, Cartago, 4 km NE Cañón Génesis II, 2350 m, collected VII.1995, P. Hanson leg., UWIM. Three females, four males (point mounted), COSTA RICA, Cartago, Dulce Nombre, Viveiro Linda Vista, 1300 m, collected VIII–X.1993, P. Hanson leg., UWIM. Two males (point mounted), COSTA RICA, Cartago, Dulce Nombre, Viveiro Linda Vista, 1400 m, collected VI–VIII.1993, P. Hanson leg., UWIM. One female, one male (point mounted), COSTA RICA, Cartago, La Cangreja, 1950 m, collected XII.1991, P. Hanson leg., UWIM. One male (point mounted), COSTA RICA, Guanacaste, Tierras Morenas, 700 m, collected III.1993, G. Rodríguez leg., UWIM. Three females (point mounted), COSTA RICA, Puntarenas, San Vito, Estac. Biol. Las Alturas, 1500 m, collected II.1992, P. Hanson leg., UWIM. One female (point mounted), COSTA RICA, Puntarenas, San Vito, Estac. Biol. Las Alturas, 1700 m, collected II–IV.1993, P. Hanson leg., UWIM. One female, one male (point mounted), COSTA RICA, Puntarenas, San Vito, Estac. Biol. Las Alturas, 1500 m, collected III.1992, P. Hanson leg., UWIM. Four females (point mounted), COSTA RICA, San Jose, 26 km N San Isidro just S of Division, 2100 m, collected II–IV.1993, P. Hanson leg., UWIM. Three females (point mounted), COSTA RICA, San José, 26 km N San Isidro just S of Division, 2100 m, collected IV–V.1993, P. Hanson leg., Malaise, UWIM. Four females (point mounted), COSTA RICA, San José, 26 km N San Isidro just S of Division, 2100 m, collected VI–VIII.1992, P. Hanson leg., Malaise, UWIM. Two females, one male (point mounted), COSTA RICA, San José, Cerro de la Muerte, 26 km N San isidro, 2100 m, collected II–V.1992, P. Hanson leg., UWIM. One female (point mounted), COSTA RICA, San José, Cerro de la Muerte, 26 km N San Isidro, 2100 m, collected II–V.1991, P. Hanson leg., UWIM. Two females (point mounted), COSTA RICA, San José, Zurqui de Moravia, 1600 m, collected III.1992, P. Hanson leg., UWIM. One male (point mounted), COSTA RICA, San Jose, Zurqui de Moravia, 1600 m, collected IV.1992, P. Hanson leg., UWIM. One female (point mounted), COSTA RICA, San José, Zurqui de Moravia, 1600 m, collected V.1992, P. Hanson leg., UWIM.

##### Distribution.

Costa Rica.

##### Biology.

Unknown.

##### Comments.

*Meteorus carolae* and *Meteorus rogerblancoi* might be confused because both share the complete occipital carina, twisted mandibles, notauli shallowly impressed and not distinct, the hind coxa strigate, tarsal claw with a large lobe, first tergite without dorsopes and ventral borders of the first tergite joined along ½ of segment. Despite their close similarity both species appear distant in the key because of the pale color on the antennae tip contrasting with dark on the rest of the structure in *Meteorus rogerblancoi* (antennae uniformly dark in *Meteorus carolae*). The pale color on the antennae tip of *Meteorus rogerblancoi* was not taking into account in the original description by Zitani et al. (1998) probably because it is too small and restricted to the last three or two flagellomeres, but the careful examination of the complete type series allows to know that it is always present in both males and females. Another constant and stable character allowing separation of both species is the hind coxa completely dark brown in *Meteorus carolae* vs. the coxa basally yellow, distally black-dark brown in *Meteorus rogerblancoi*. On the couplet 44 of the key *Meteorus carolae* matches closely to *Meteorus martinezi*. They have in common the same set of features share between *Meteorus carolae* and *Meteorus rogerblancoi*, but *Meteorus carolae* has the coxa dark brown (hind coxa dorsally dark brown, ventrally yellow in *Meteorus martinezi*), antennae with 24–27 flagellomeres (antennae with 31 flagellomeres in *Meteorus martinezi*) and the convergent eyes in frontal view, face maximum width/minimum width = 1.4–1.6 (face maximum width/minimum width = 1.1 in *Meteorus martinezi*).

##### Etymology.

*Meteorus carolae* is named after Mrs. Carol Abram, Scott Shaw’s sister. Thank you for teaching me to read, and encouraging my entomological pursuits.

#### Meteorus eurysaccavorus

Taxon classificationAnimaliaHymenopteraBraconidae

Aguirre, Almeida & Shaw sp. n.

http://zoobank.org/C97793CA-C8CF-4806-B744-D248820706AA

Figures 83–90

##### Diagnosis.

Occipital carina complete, ocelli small (ocelli ocular distance 2.7–3 × ocellar diameter), posterior area of temple and gena coriaceous, eyes convergent (face width 1.6 × minimum face width), mandibles untwisted, notauli distinct, lateral lobes of mesoscutum coriaceous, propodeum carinate-rugose, transverse carina on propodeum present, vein 3RSb distinctly curved, marginal cell short, dorsope and laterope present; ventral borders of first tergite widely separated, basal area of T3 coriaceous, ovipositor long (ovipositor 2.4 × longer than first tergite).

**Figures 83–90.**
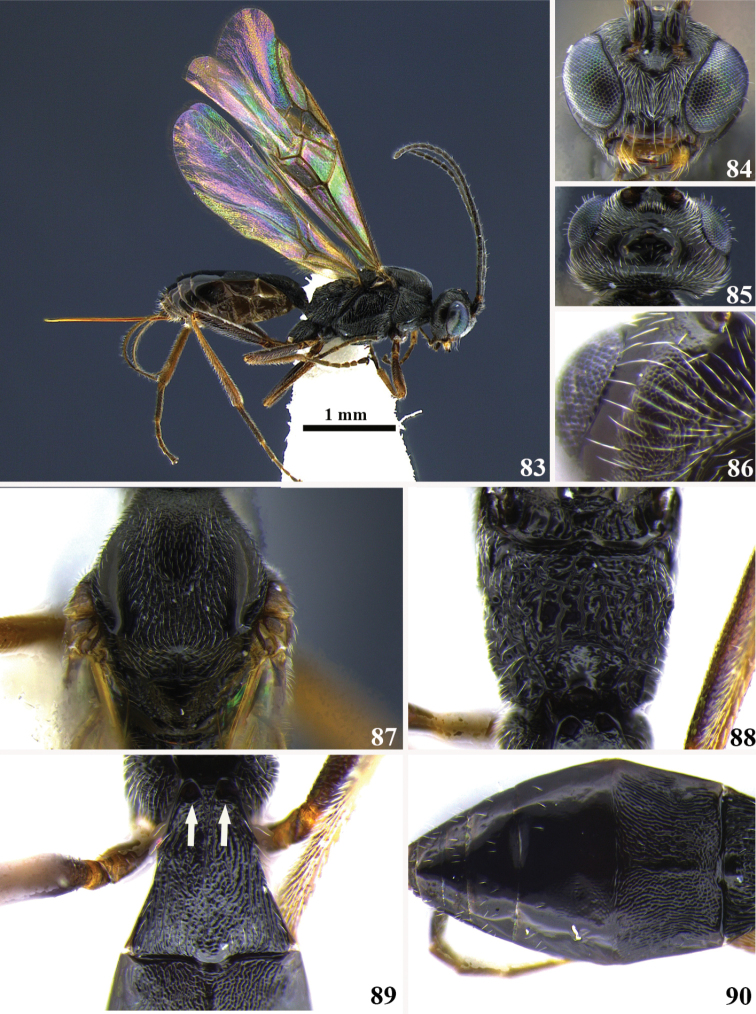
*Meteorus eurysaccavorus* sp. n. female. **83** Habitus in lateral view **84** head in frontal view **85** head in dorsal view **86** temple in posterior view **87** mesonotum in dorsal view **88** propodeum in dorsal view **89** first tergite in dorsal view, the arrows indicate the dorsopes’ location **90** metasoma, excluding the first tergite, in dorsal view.

##### Body color.

Mostly black except: prothoracic legs brown from trochanter along tarsus; mesothoracic and metathoracic legs with trochanter, trochantellus, femur and tarsus dark brown, tibia light brown; sterna dark brown; wings hyaline.

Body length 3.4 mm.

##### Head.

Antenna with 19 flagellomeres; flagellar length/width ratios as follows: F1 = 5.5, F2 = 3.7, F3 = 3.7, F17 = 1.7, F18 = 1.7, F19 = 2.7; head 1.2 wider than high; occipital carina complete; ocelli ocular distance 3 × ocellar diameter; head height 1.5 × eye height; temples length 0.6 × eyes length in dorsal view; vertex in dorsal view not descending vertically behind the lateral ocelli; posterior area of temple and gena coriaceous; frons puncticulate; eyes convergent, maximum face width 1.6 × minimum face width; face finely rugulose; minimum face width 0.8 × clypeus width; clypeus smooth and polished; malar space length 0.6 × mandible width basally; mandibles untwisted.

##### Mesosoma.

Pronotum in lateral view completely rugose; propleuron mostly smooth except rugulose on the anterior part; notauli distinctive and rugose; mesonotal lobes well defined; lateral lobes of mesoscutum coriaceous; scutellar furrow with one distinctive carina; mesopleuron mostly smooth but rugulose close to tegula; precoxal sulcus long, wide and rugose-costate; metapleuron rugose; propodeum carinate-rugose; transversal carina on propodeum present; median depression on propodeum absent.

##### Legs.

Hind coxa strigate; tarsal claw simple.

##### Wings.

Wing length 3.2 mm; second submarginal cell of forewing not strongly narrowed anteriorly; vein r 0.6 × length of 3RSa; vein 3RSb distinctly curved; marginal cell short; vein 3RSa 0.7 × length of rm; vein m-cu of forewing antefurcal; vein 1M 1.1 × length of cu-a; vein 1M 0.6 × length of 1r-m.

##### Metasoma.

Dorsope and laterope present; ventral borders of first tergite widely separated; first tergite costate-rugulose; T2 coriaceous-costate, costae divergent; basal area of T3 coriaceous; ovipositor long and straight, ovipositor 2.4 × longer than first tergite.

##### Cocoon.

Unknown.

##### Female variation.

Body length 3.3–3.5 mm; antenna with 19–20 flagellomeres; ocelli ocular distance 2.7–3 × ocellar diameter; temples length 0.6–0.7 × eyes length in dorsal view; frons finely rugulose or puncticulate; minimum face width 0.7–0.8 × clypeus width; malar space length 0.5–0.6 × mandible width basally; scutellar furrow with four clearly distinctive carinae; precoxal sulcus rugose-costate or rugose-colliculate; wing length 3.2–3.4 mm; vein r 0.6–0.9 × length of 3RSa; vein 3RSa 0.7–0.8 × length of rm; vein 1M 0.9–1.1 × length of cu-a; vein 1M 0.6–0.8 × length of 1r-m; first tergite costate-rugulose, or entirely rugulose; ovipositor 2.1–2.4 × longer than first tergite.

##### Male variation.

Body length 3.4–3.5 mm; antenna with 23–24 flagellomeres; head height 1.6–1.7 × eye height; temple length 0.8–0.9 × eye length in dorsal view; maximum face width 1.2–1.3 × minimum face width; minimum face width 0.8–1 × clypeus width; malar space length 0.6–0.8 × mandible width basally; propleuron smooth and polished; scutellar furrow with six clearly distinctive carinae; wing length 3 mm; vein r 0.6 × length of 3RSa; vein 3RSa 0.8–0.9 × length of rm; vein 1M 1.1–1.3 × length of cu-a; vein 1M 0.6–0.7 × length of 1r-m; first tergite rugose.

##### Type locality.

BOLIVIA, La Paz, Patacayama Research Station.

##### Type specimen.

Holotype female (point mounted). Original label: BOLIVIA, La Paz, Patacayama Research Station, collected II–III.1995. Reared from larvae of *Eurysacca melanocampta* Meyrick, UWIM.

Paratypes. Two females and two males (point mounted), same data as the holotype, UWIM.

##### Distribution.

BOLIVIA, La Paz, Patacayama Research Station.

##### Biology.

Parasitoid of *Eurysacca melanocampta* (Gelechiidae).

##### Comments.

*Meteorus eurysaccavorus* is the only Neotropical *Meteorus* species with a combination of coriaceous sculpture on temple, gena, mesonotum and T2, presence of dorsopes on the first metasomal tergite, and the vein 3RSb of the frontal wing distinctly curved (such a vein is entirely straight in the rest of species). When *Meteorus eurysaccavorus* is compared with the previously known Neotropical *Meteorus*, the morphologically most-similar species is *Meteorus muiscai*, since both of them share a complete occipital carina, simple tarsal claw, metapleuron rugose and presence of dorsopes. However, *Meteorus muiscai* is completely smooth and shiny on the body surfaces on which *Meteorus eurysaccavorus* displays coriaceous sculpture, and the legs of *Meteorus eurysaccavorus* are dark brown to black, in contrast to yellow in *Meteorus muiscai*.

##### Etymology.

The specific epithet is composed by the stem *eurysacca* after the host genus name, and the suffix “vorus” derived from the latin “vor” that means voracious, referring to the feeding habit of the wasp larva on this gelechiid caterpillar.

#### Meteorus fallacavus

Taxon classificationAnimaliaHymenopteraBraconidae

Aguirre, Almeida & Shaw sp. n.

http://zoobank.org/6F771503-FAC3-4E1D-A104-6359390BD2B8

Figures 91–97

##### Diagnosis.

Occipital carina complete, mandibles twisted, notauli deeply impressed, distinctive and rugose-foveate, first tergite laterally flattened, hind coxa strigate-rugulose; tarsal claw with a large lobe, a couple of cavities (false dorsopes) on the first tergite between the basal extreme and the spiracles, first tergite laterally flattened; ventral borders of first tergite touching distally for a short distance, ovipositor 2.0–2.2 × longer than first tergite.

**Figures 91–97.**
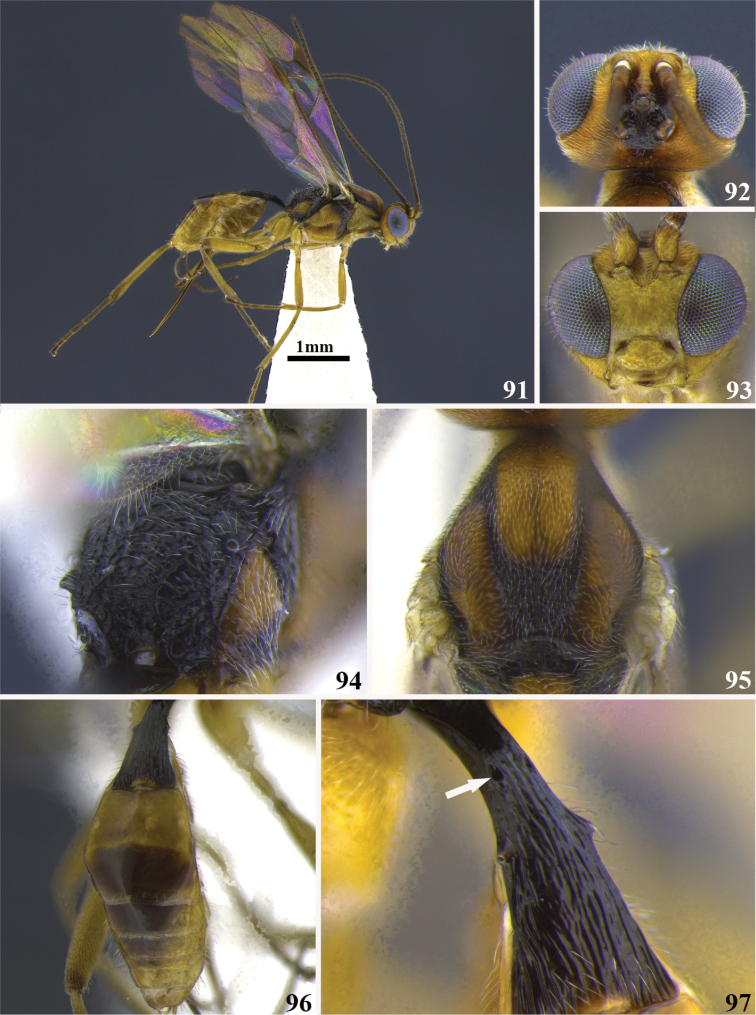
*Meteorus fallacavus* sp. n. female. **91** Habitus in lateral view **92** head in dorsal view **93** head in frontal view **94** propodeum in dorso-lateral view **95** mesoscutum in dorsal view **96** metasoma in dorsal view **97** first tergite in dorso-lateral view, the arrow indicates the position of the “false” dorsope.

##### Body color.

Antenna dark brown; annulus absent; face, clypeus and gena yellow; frons, temple and vertex orange; area between ocelli and occiput black. Anterior half of propleuron brown, posterior half yellow; pronotum yellow; mesonotal lobes and scutellum brown, notauli and area among lobes black; mesopleuron brown except dorsal and anterior borders black; metanotum totally black; metapleuron brown except ventral border black; propodeum black. Pro and mesothoracic legs yellow except tarsus brown; metathoracic legs yellow except tibia apically and tarsus dark brown. T1 black, T2 yellow, remaining terga brown; sterna light brown. Wing membrane hyaline, stigma brown.

##### Body length.

3.9 mm.

##### Head.

Antenna with 27 flagellomeres; flagellar length/width ratios as follows: F1 = 4.1, F2 = 3.5, F3 = 3, F25 = 1.7, F26 = 1.7, F27 = 2.7; head 1.2 wider than high; occipital carina complete; ocellus-ocullar distance 1.2 × ocellar diameter; head height 1.4 × eye height; temple length 0.4 × eye length in dorsal view; vertex in dorsal view not descending vertically behind the lateral ocelli; frons smooth and polished; face maximum width 1.3 × minimum width; face punctate; face minimum width equal to clypeus width; clypeus rugulose; malar space length 0.5 × mandible width basally; mandibles twisted.

##### Mesosoma.

Pronotum in lateral view coarsely rugulose; propleuron slightly puncticulate; notauli deeply impressed, distinctive and rugose-foveate; mesonotal lobes well defined; central lobe of mesoscutum punctate; scutellar furrow with three carinae; mesopleuron mostly puncticulate, rugose close to the tegula; precoxal sulcus long, narrow and rugose-foveate; metapleuron mostly smooth, rugose close to the coxa; propodeum rugose and devoid of both carinae and a median depression.

##### Legs.

Hind coxa strigate-rugulose; tarsal claw with a large lobe.

##### Wings.

Wing length 3.4 mm; second submarginal cell of forewing not strongly narrowed anteriorly. Front wing: length of vein r 0.4 × length of vein 3RSa; vein 3RSb straight; vein m-cu of forewing intersticial. Hind wing: length of vein 1M 1.2 × length of vein cu-a; length of vein 1M equal to length of vein r-m.

##### Metasoma.

Dorsope present, very small (actually it is a false dorsope, see explanation on comments below); first tergite laterally flattened; ventral borders of first tergite touching distally for a short distance; first tergite with costae parallel faintly demarcated; ovipositor thickened basally and straight; ovipositor 2.2 × longer than first tergite.

##### Cocoon.

Unknown.

##### Female variation.

Propleuron yellow except lateral and anterior borders brown; median mesonotal lobe and scutellum testaceous, lateral mesonotal lobes dark brown, notauli and area between mesonotal lobes black; mesopleuron orange except dorsal and anterior borders black; metapleuron orange except ventral border black; prothoracic legs completely yellow; mesothoracic legs with coxa, trochanter and trochantellus white, remaining dark brown; antenna with 26 flagellomeres; ocellus-ocullar distance 1.1–1.4 × ocellar diameter; head height 1.5 × eye height; metapleuron rugulose; ovipositor 2.0–2.2 × longer than first tergite.

##### Male variation.

Unknown.

##### Type locality.

COSTA RICA, Puntarenas, San Vito, Estación Biológica Las Alturas, 1500 m.

##### Type specimen.

Holotype female (point mounted). COSTA RICA, Puntarenas, San Vito, Estación Biológica Las Alturas, 1500 m, collected XII.1991, Paul Hanson leg., UWIM.

Paratypes. One female (point mounted), COSTA RICA, Puntarenas, San Vito, Estación Biológica Las Alturas, 1500 m, collected I.1992, Paul Hanson leg., UWIM. One female (point mounted), COSTA RICA, Cartago, 4 Km NE cañón Génesis II, 2350 m, collected IX.1996, P. Hanson leg., UWIM.

##### Distribution.

Costa Rica, at the provinces of Cartago and Puntarenas.

##### Biology.

Unknown.

##### Comments.

*Meteorus fallacavus* displays a distinctive pair of holes on the first metasomal tergite, ahead of the spiracles. In a strict sense these are not dorsopes because the presence of dorsopes always is correlated with ventral borders of the first tergite widely separated as remarked by Muesebeck (1923), Nixon (1941), Huddleston (1980) and corroborated in the Neotropical fauna (Aguirre et al. 2011). *Meteorus fallacavus* has the ventral borders of the first tergite basally separated but distally touching by a short distance, feature allowing separate it from *Meteorus magdalensis*, its most similar congeneric species, which displays a true pair of dorsopes together with ventral borders of the first tergite widely separated. Both species have the notauli deeply impressed and distinct, as well as the first metasomal tergite unicolored, but *Meteorus magdalensis* is mostly black while *Meteorus fallacavus* is mostly yellow with black areas dorsally. Moreover, *Meteorus fallacavus* might be distinguished by having twisted mandibles (untwisted in *Meteorus magdalensis*), tarsal claw with a large lobe (tarsal claw simple in *Meteorus magdalensis*) and the vertex in lateral view flattened (vertex convex in lateral view in *Meteorus magdalensis*).

##### Etymology.

The specific epithet is composed by the latin prefix “falla” which means false and “cavus” meaning cavity, since the pseudodorsope is the most distinctive feature for this species.

#### Meteorus flavistigma

Taxon classificationAnimaliaHymenopteraBraconidae

Aguirre, Almeida & Shaw sp. n.

http://zoobank.org/894CEC06-624C-4F74-9A3E-B2E0D09DFA2D

Figures 98–103

##### Diagnosis.

Occipital carina complete; ocelli small, ocellus-ocullar distance 1.4–1.8 × ocellar diameter; mandibles moderately twisted; notauli deeply impressed, distinctive and foveolate; propodeum aerolate-rugose; hind coxa punctate and polished; tarsal claw with large lobe; dorsope absent; T1 laterally flattened; ventral borders of first tergite separated basally and joined apically along almost ½ of segment; ovipositor 2.5 × longer than first tergite; stigma yellow.

**Figures 98–103.**
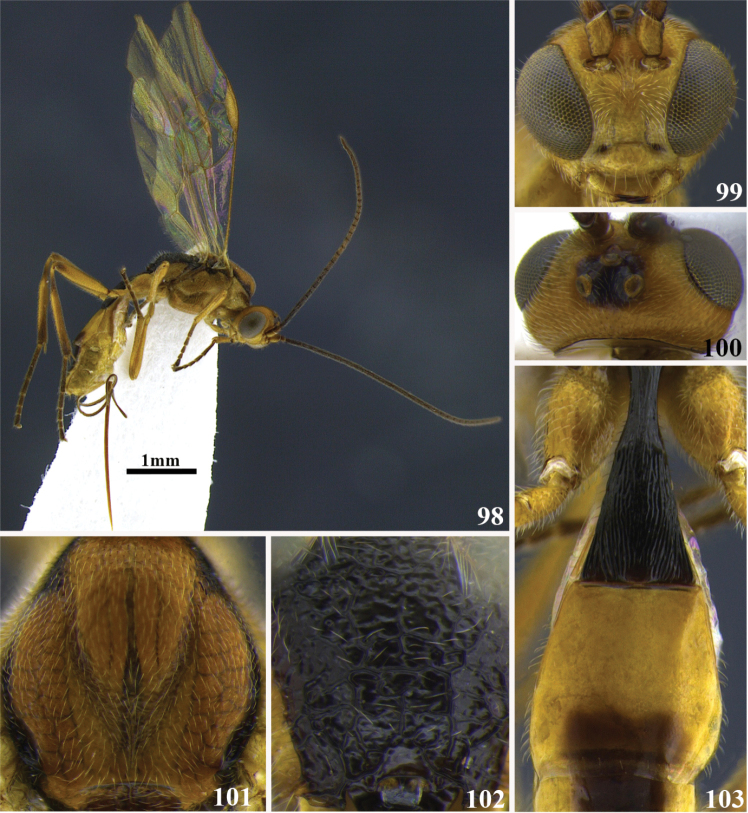
*Meteorus flavistigma* sp. n. female. **98** Habitus lateral view **99** head in frontal view **100** head in dorsal view **101** mesoscutum in dorsal view **102** propodeum in posterior view **103** metasoma in dorsal view.

##### Body color.

Antenna dark brown, annulus absent; head orange except area between ocelli black. Propleuron orange; pronotum either testaceous or yellow; mesonotum orange, bordered by a black strip; mesopleuron orange-testaceous; metanotum black dorsally, orange and black laterally; metapleuron either testaceous or yellow; propodeum black. Prothoracic legs testaceous; mesothoracic legs testaceous; metathoracic legs testaceous except coxa apically, tibia and tarsus dark brown. T1 black; T2–T7 with a large dorso-medial dark brown oval-shaped area surrounded by yellow; sterna yellow. Wing membrane hyaline; stigma yellow.

##### Body length.

4 mm.

##### Head.

Antenna with 26 flagellomeres; flagellar length/width ratios as follows: F1 = 3.7, F2 = 4, F3 = 3.1, F24 = 1.7. F25 = 1.5. F26 = 2.3; head 1.2 wider than high; occipital carina complete; ocellus-ocullar distance 1.4 × ocellar diameter; head height 1.8 × eye height; temple length 0.5 × eye length in dorsal view; vertex in dorsal view not descending vertically behind the lateral ocelli; frons surface irregular; face maximum width 1.3 × minimum width; face punctuate; face minimum width 0.8 × clypeus width; clypeus smooth with dispersed punctures; malar space length 0.5 × mandible width basally; mandibles moderately twisted.

##### Mesosoma.

Pronotum in lateral view carinated; propleuron puncticulate and shiny; notauli deeply impressed, distinctive and foveolate; mesonotal lobes well defined; central lobe of mesoscutum punctuate; scutellar furrow with three carinae; mesopleuron punctate; precoxal sulcus short, narrow and foveate; metapleuron surface irregular and polished except either rugose or finely rugulose close to the coxa; propodeum aerolate-rugose, without a median depression, transversal or longitudinal carinae.

##### Legs.

Hind coxa punctate and polished; tarsal claw with large lobe.

##### Wings.

Wing length 3.6 mm; second submarginal cell of forewing not strongly narrowed anteriorly. Front wing: length of vein r 0.8 × length of vein 3RSa; vein 3RSb straight; length of vein 3RSa equal to length of vein r-m; vein m-cu antefurcal. Hind wing: length of vein 1M 1.2 × length of vein cu-a; length of vein 1M equal to length of vein r-m.

##### Metasoma.

Dorsope absent; T1 laterally flattened; ventral borders of first tergite separated basally and joined apically along almost ½ of segment; first tergite with costae almost parallel; ovipositor thickened basally and straight; ovipositor 2.5 × longer than first tergite; T2–T7 smooth.

##### Cocoon.

Unknown.

##### Female variation.

T2 yellow, T3 brown, T4–T6 brown medially and yellow laterally, T7–T8 yellow; body length 4.2 mm; ocellus-ocullar distance 1.8 × ocellar diameter; head height 1.5 × eye height; temple length 0.4 × eye length in dorsal view; frons smooth and polished; face maximum width 1.5 × minimum width; malar space length 0.6 × mandible width basally; pronotum in lateral view foveate, rugose or rugose-carinate, notauli rugose-foveate, scutellar furrow with four carinae; metapleuron dorsally punctate and ventrally foveate; wing length 3.7 mm; first tergite with costae convergent posteriorly.

##### Male variation.

Both lateral mesonotal lobes and the median one apically black, yellow the rest; mesopleuron either yellow except area close to the tegula dark brown or orange on the middle, black dorsally and ventrally; pro and mesothoracic legs yellow except tarsus brown; metathoracic legs yellow except tibia brown, femur apically and tarsus dark brown; T2 basally yellow-orange, remaining dark brown; body length 3.8 mm; antenna with 32 flagellomeres; ocellus-ocullar distance equal to ocellar diameter; wing length 3.4 mm; front wing: length of vein r 0.6 × length of vein 3RSa; first tergite costate-reticulate.

##### Type locality.

COSTA RICA, San José, Cerro de la Muerte, 19 Km South, 3 Km West, Empalme, 2600 m.

##### Type specimen.

Holotype female (point mounted), COSTA RICA, San José, Cerro de la Muerte, 19 Km South, 3 Km West, Empalme, 2600 m, collected XII.1992, P. Hanson leg., UWIM.

Paratypes. Three females and one male (point mounted), COSTA RICA, Heredia, Estación Barva, Parque Natural Braulio Carillo, 2500 m, collected V.1990, A. Fernández leg., UWIM. One male (point mounted), COSTA RICA, Heredia, Estación Barva, Parque Natural Braulio Carillo, 2500 m, collected VI.1990, B. Apu and G. Varela leg., UWIM. One male (point mounted), COSTA RICA, Puntarenas, San Vito, Estación Biológica Las Alturas, 1500 m, collected II.1992, P. Hanson leg., UWIM.

##### Distribution.

Costa Rica, at the provinces of San Jose, Heredia, and Puntarenas.

##### Biology.

Unknown.

##### Comments.

*Meteorus flavistigma* shares with *Meteorus boyacensis* the mandibles moderately twisted and ventral borders of the first tergite basally separated and joined along the rest of the segment. *Meteorus flavistigma* might be distinguished from *Meteorus boyacensis* by the tarsal claw with a large lobe (tarsal claw simple in *Meteorus boyacensis*), and body mostly yellow except mesosoma and metasoma with dark areas (completely black-dark brown in *Meteorus boyacensis*).

##### Etymology.

This species is so-named because of the yellow stigma on the front wing: “flavis” is the Latin prefix meaning yellow.

#### Meteorus haimowitzi

Taxon classificationAnimaliaHymenopteraBraconidae

Aguirre, Almeida & Shaw sp. n.

http://zoobank.org/9EE42698-A0C2-4796-99D5-C8B40BF6EFC4

Figures 104–110

##### Diagnosis.

Occipital carina complete; large ocelli, ocellus-ocullar distance 0.3 × ocellar diameter; large ayes, head height 1.3 × eye height; malar space very short, malar space length 0.1 × mandible width basally; mandibles twisted; notauli shallow, not distinctive and rugose; hind coxa strigate; tarsal claw with large lobe; dorsope absent; ventral borders of first tergite joined completely along ½ of segment; mesopleuron completely yellow; metanotum dorsally brown, yellow laterally.

**Figures 104–110.**
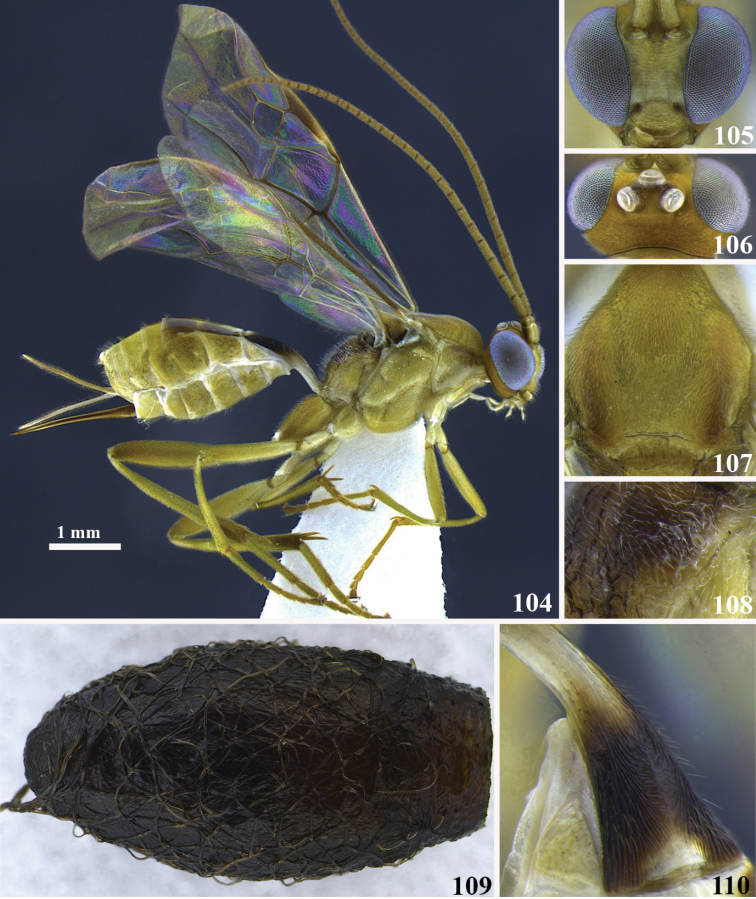
*Meteorus haimowitzi* sp. n. female. **104** Habitus in lateral view **105** head in frontal view **106** head in dorsal view **107** mesoscutum in dorsal view **108** propodeum in postero-lateral view **109** cocoon **110** first tergite in dorso-lateral view.

##### Body color.

Antenna, face and clypeus yellow; annulus absent; remaining head orange. Propleuron, pronotum, mesopleuron and metapleuron yellow; mesonotum yellow except a couple of faint light brown patches on each lateral mesonotal lobe; metanotum dorsally brown, yellow laterally; propodeum light brown. Pro and metathoracic legs yellow; mesothoracic coxa, trochanter and trochantellus white, remaining leg dark brown. T1 having the basal half and a narrow patch along the distal border yellow, medially black; a median white-yellow broad hourglass-shaped pattern on T2, T3 brown, T4–T8 yellow; sterna yellow. Wing membrane hyaline; stigma brown.

##### Body length.

5.7 mm.

##### Head.

Antenna with 31 flagellomeres; flagellar length/width ratios as follows: F1 = 3.6, F2 = 3.3, F3 = 2.8, F29 = 2, F30 = 1.7, F31 = 3.3; head 1.2 wider than high; occipital carina complete; ocellus-ocullar distance 0.3 × ocellar diameter; head height 1.3 × eye height; temple length 0.6 × eye length in dorsal view; vertex in dorsal view descending vertically behind the lateral ocelli; frons smooth and polished; face maximum width 1.4 × minimum width; face strigulate; face minimum width 0.8 × clypeus width; clypeus strigulate; malar space length 0.1 × mandible width basally; mandibles twisted.

##### Mesosoma.

Pronotum in lateral view carinate-rugose; propleuron rugulose-costate, with costae divergent posteriorly; notauli shallow, not distinctive and rugose; mesonotal lobes not well defined; central lobe of mesoscutum punctate; scutellar furrow with three carinae; mesopleuron punctate; precoxal sulcus long, narrow and carinate-rugose; most metapleuron surface smooth and polished except irregular to rugose close to the hind coxa; propodeum rugose and devoid of both longitudinal and transversal carinae, median depression absent.

##### Legs.

Hind coxa strigate; tarsal claw with large lobe.

##### Wings.

Wing length 5.3 mm; second submarginal cell of forewing not strongly narrowed anteriorly. Front wing: length of vein r 0.3 × length of vein 3RSa; vein 3RSb straight; length of vein 3RSa 1.2 × length of vein r-m; vein m-cu antefurcal. Hind wing: length of vein 1M 0.9 × length of vein cu-a; length of vein 1M 0.8 × length of vein r-m.

##### Metasoma.

Dorsope absent; ventral borders of first tergite joined completely along ½ of segment; first tergite with costae convergent posteriorly; ovipositor thickened basally and straight; ovipositor 1.4 × longer than first tergite.

##### Cocoon.

Length 6.6 mm; width 2.8 mm; black-dark brown, loosely wrapped by its silk; the edge of the emergence hole is rough, the cap is missing. The thread is approximately 36 mm long.

##### Female variation.

Unknown.

##### Male variation.

Unknown.

##### Type locality.

COSTA RICA, Heredia, Vara Blanca, 2000 m.

##### Type specimen.

Holotype female (point mounted), COSTA RICA, Heredia, Vara Blanca, 2000 m, collected IV.27.2002, Kenji Nishida leg., UWIM.

Paratype. Unknown.

##### Distribution.

Costa Rica, Province of Heredia.

##### Biology.

Solitary parasitoid reared from its cocoon.

##### Comments.

*Meteorus haimowitzi* and *Meteorus imaginatus* Jones share more morphological features between them than with any other species in the genus; the most relevant are: big eyes, head height 1.3 × or less eye height, occipital carina complete, mandibles completely twisted, notauli shallow and not distinct, tarsal claw with a large lobe, first metasomal tergite without dorsopes and ventral borders of first tergite completely joined along ½ of segment. *Meteorus hamowitzi* differs from *Meteorus imaginatus* by the metanotum dorsally black-dark brown and laterally yellow (metanotum completely black-dark brown in *Meteorus imaginatus*), hind legs yellow (hind legs dark brown in *Meteorus imaginatus*) and mesonotal lateral lobes mostly yellow (mesonotal lateral lobes dark brown in *Meteorus imaginatus*). Interestingly another conspicuous character to distinguish both species is in the cocoon, which is ornamented with a crown-like silk arrangement nearby the opening apex in *Meteorus imaginatus*, but this is absent in *Meteorus haimowitzi* (see Jones and Shaw 2012, p. 10, fig. 21).

##### Etymology.

This species is named after our entomologist colleague and parasitoid-lover Larry Haimowitz.

#### Meteorus magnoculus

Taxon classificationAnimaliaHymenopteraBraconidae

Aguirre, Almeida & Shaw sp. n.

http://zoobank.org/734B83C5-1DCD-4CAC-ABB6-817BD179B3AA

Figures 111–117 , 118–120

##### Diagnosis.

Occipital carina complete; large ocelli, ocellus-ocullar distance 0.5–0.6 × ocellar diameter; huge eyes, head height 1.2–1.4 × eye height; mandibles twisted; notauli deeply impressed, distinctive and rugose; propodeum aerolate-rugose; dorsope absent; ventral borders of first tergite joined completely along ½ of segment; ovipositor basally thickened and slightly curved; ovipositor 2.4–3 × longer than first tergite; mesosoma ferruginous, head mostly dark, metasoma and legs white and black.

**Figures 111–117.**
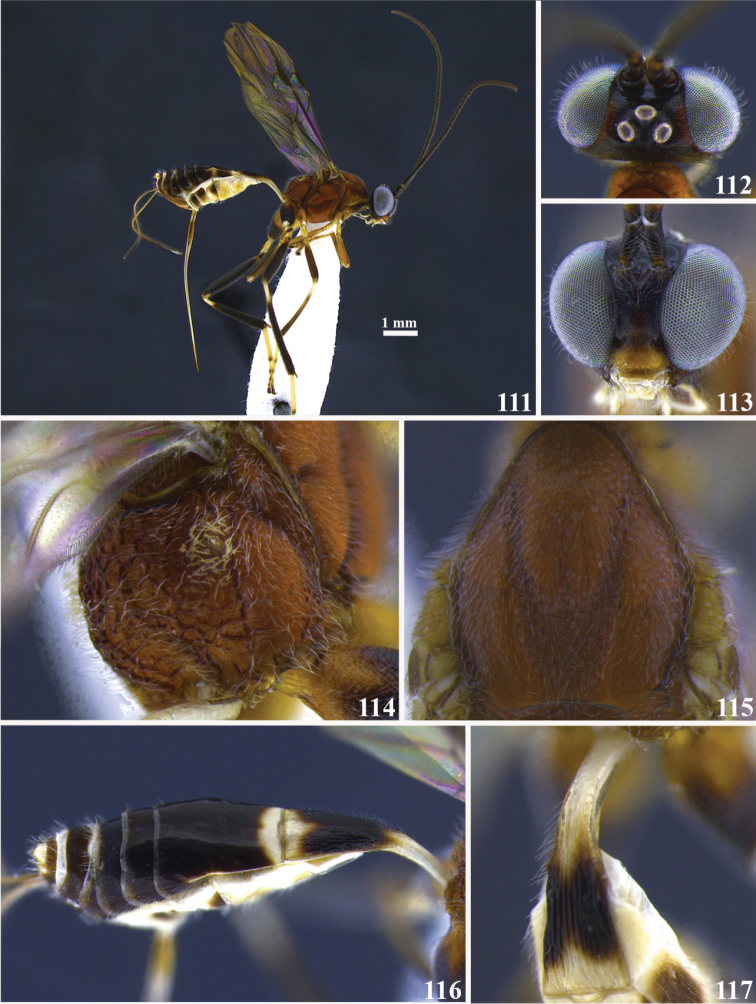
*Meteorus magnoculus* sp. n. female. **111** Habitus in lateral view **112** head in dorsal view **113** head in frontal view **114** propodeum in dorso-lateral view **115** mesoscutum in dorsal view **116** metasoma in dorso-lateral view **117** first tergite in dorso-lateral view.

**Figures 118–120.**
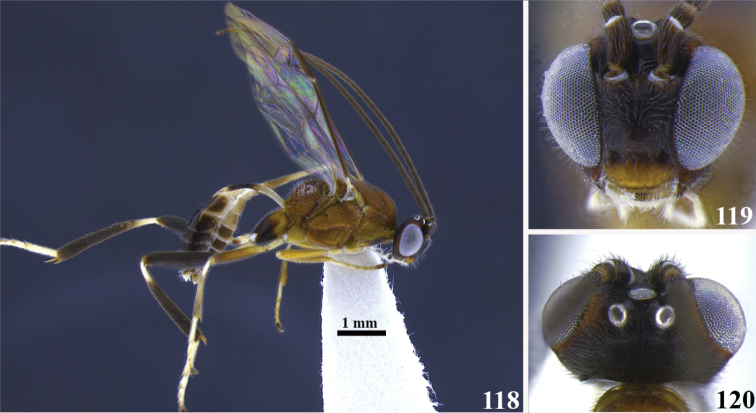
*Meteorus magnoculus* sp. n. male. **118** Habitus lateral view **119** head in frontal view **120** head in dorsal view.

##### Body color.

Antenna dark brown; annulus absent; head black except a small brown patch between each lateral ocelli and its closest compound eye; clypeus yellow; mesosoma mostly ferruginous except propleuron anterior 2/3 black, posterior 1/3 and interior borders yellow; pronotum ferruginous on the upper half, then gradually becomes yellow toward the lower border. Prothoracic coxa, trochanter and trochantellus yellow, remaining leg orange; mesothoracic legs brown except coxa, trochanter, trochantellus, both femur and tibia basally, and most of tarsus yellow. Metathoracic coxa basally orange-ferruginous, distally black; metathoracic trochanter, tibia basally and tarsus white-yellow; remaining hind leg black. Basal half and a narrow patch along the distal border of T1 yellow, T1 medially black; T2 on the basal border and T7 throughout white-yellow, remaining T2 and T3–T5 black, T6 and T8 brown; sterna yellow white, with brown patches on the sterna 5–7. Wings hyaline; stigma dark brown.

##### Body length.

6.6 mm.

##### Head.

Antenna with 33 flagellomeres; flagellar length/width ratios as follows: F1 = 4.2, F2 = 4, F3 = 3.3, F31 = 2.2, F32 = 2, F33 = 3; head 1.2 wider than high; occipital carina complete; ocellus-ocullar distance 0.6 × ocellar diameter; huge eyes, head height 1.2 × eye height; temple length 0.3 × eye length in dorsal view; vertex in dorsal view not descending vertically behind the lateral ocelli; frons smooth and polished; face maximum width 1.5 × minimum width; face puncticulate; face minimum width 0.7 × clypeus width; clypeus punctate; malar space length 0.1 × mandible width basally; mandibles twisted.

##### Mesosoma.

Pronotum in lateral view carinate and rugose; propleuron coarsely rugose; notauli deeply impressed, distinctive and rugose; mesonotal lobes well defined; central lobe of mesoscutum punctulate; scutellar furrow with three carinae; mesopleuron punctate; precoxal sulcus long, narrow and aerolate-rugose; metapleuron rugose; propodeum aerolate-rugose, longitudinal and transversal carinae absent, median depression weakly impressed.

##### Legs.

Hind coxa strigate and punctate; tarsal claw with a large lobe.

##### Wings.

Wing length 4.9 mm; second submarginal cell of forewing not strongly narrowed anteriorly. Front wing: length of vein r 0.5 × length of vein 3RSa; vein 3RSb straight; length of vein 3RSa 0.9 × length of vein r-m; vein m-cu antefurcal. Hind wing: length of vein 1M 1.2 × length of vein cu-a; length of vein 1M 1.1 × length of vein r-m.

##### Metasoma.

Dorsope absent; ventral borders of first tergite joined completely along ½ of segment; first tergite with faintly demarcate and parallel costae; ovipositor basally thickened and slightly curved; ovipositor 2.9 × longer than first tergite.

##### Cocoon.

Unknown.

##### Female variation.

Body length 6 mm; antenna with 35–36 flagellomeres; ocellus-ocullar distance 0.5 × ocellar diameter; head height 1.3–1.4 × eye height; temple length 0.4 × eye length in dorsal view; face maximum width 1.4 × minimum width; face minimum width 0.8–0.9 × clypeus width; malar space length 0.2 × mandible width basally; wing length 4.8 mm. Front wing: length of vein r 0.4 × length of vein 3RSa; length of vein 3RSa 1.2 × length of vein r-m. Hind wing: length of vein 1M 1.1–1.3 × length of vein cu-a; length of vein 1M 1–1.4 × length of vein r-m; ovipositor 2.4–3 × longer than first tergite.

##### Male variation.

T2 with a yellow cup-shape area basally, remaining black; sterna 2–3 yellow-cream, sterna 4–8 brown; wings hyaline; body length 5.2 mm; antenna with 32 flagellomeres; head 1.1 wider than high; ocellus-ocullar distance equal to ocellar diameter; head height 1.4 × eye height; temple length 0.5 × eye length in dorsal view; frons strigulate; face maximum width 1.1 × minimum width; face strigate-punctate; face minimum width 0.9 × clypeus width; malar space length 0.4 × mandible width basally; wing length 4.1 mm; length of vein 3RSa equal to length of vein r-m; vein m-cu of forewing intersticial; length of vein 1M 0.9 × length of vein r-m; first tergite with costae parallel.

##### Type locality.

COSTA RICA, San Jose, San Pedro, Sabanilla.

##### Type specimen.

Holotype female (point mounted), COSTA RICA, San Jose, San Pedro, Sabanilla, collected from a pyralid leaf folder on *Ipomea* [correct spelling *Ipomoea*, A/N] XI.1997, X. Miranda leg., UWIM.

Paratype. One female, one male, same data as holotype, UWIM.

##### Distribution.

Costa Rica, province of San Jose.

##### Biology.

Parasitoid of a leaf folder pyralid (Lepidoptera: Pyralidae) sampled on *Ipomoea* (Convolvulaceae).

##### Comments.

Both the big eyes and large and colorful body make *Meteorus magnoculus* very distinct from the other species of the genus. The most similar one is *Meteorus cecavorum* sharing with *Meteorus magnoculus* the occipital carina complete, mandibles totally twisted, first metasomal tergite without dorsopes and ventral borders of first tergite joined along ½ of segment. But *Meteorus magnoculus* is easy to separate by its bigger eyes (head height/eye height = 1.3–1.4 vs. 1.5–1.6 in *Meteorus cecavorum*), bigger ocelli (ocellus-ocullar distance/ocellar diameter = 0.5–0.6 vs. 1.2–1.6 in *Meteorus cecavorum*) shorter malar space (malar space length/mandible width basally = 0.1 vs. 0.8–1.2 in *Meteorus cecavorum*) and its combination of ferruginous, black and white on the body (mostly black-dark brown in *Meteorus cecavorum*).

##### Etymology.

*Meteorus magnoculus* is, until now, the *Meteorus* species with biggest relative eye size inhabiting the Neotropical Region. The specific epithet is composed by the Latin prefix “magno” meaning large, and the Latin root “oculus” meaning eye.

#### Meteorus martinezi

Taxon classificationAnimaliaHymenopteraBraconidae

Aguirre, Almeida & Shaw sp. n.

http://zoobank.org/DFD2471B-3FD0-40F4-848D-D8645FC4F4FF

Figures 121–127

##### Diagnosis.

Occipital carina complete; face parallel in frontal view, face maximum width 1.1 × minimum width; mandibles twisted; notauli shallow, not distinctive and rugose; hind coxa strigate; tarsal claw with large lobe; dorsope absent; ventral borders of first tergite joined completely along ½ of segment; ovipositor 2.3 × longer than first tergite; body mostly dark.

**Figures 121–127.**
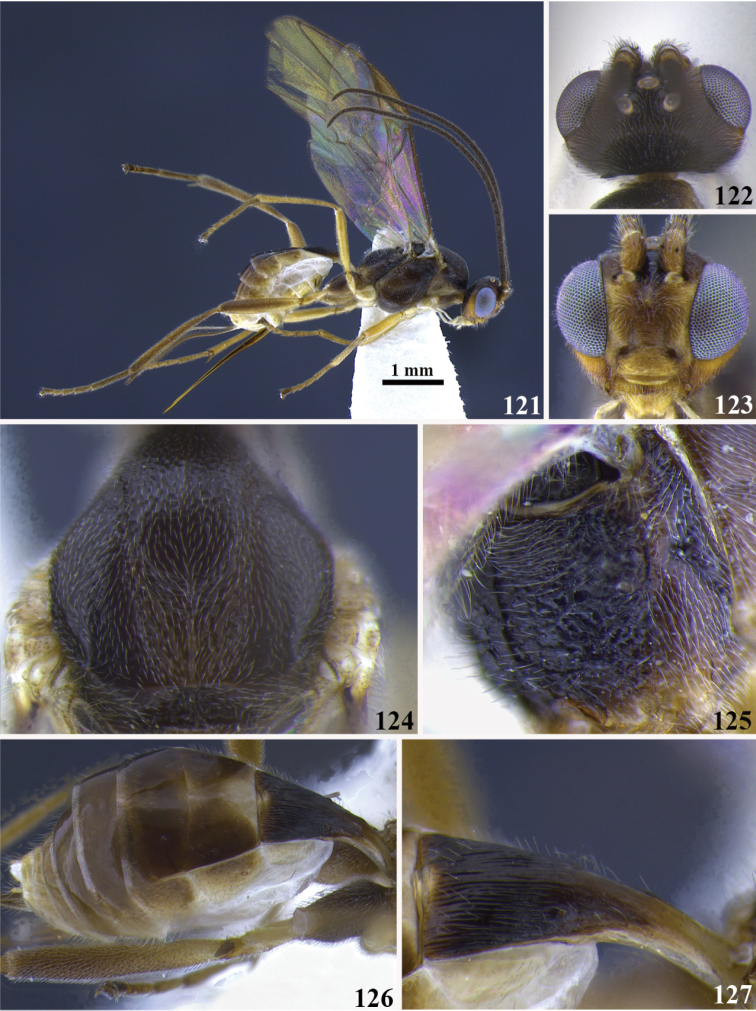
*Meteorus martinezi* sp. n. female. **121** Habitus in lateral view **122** head in frontal view **123** head in dorsal view **124** mesoscutum in dorsal view **125** propodeum in dorso-lateral view **126** metasoma in dorso-lateral view **127** first tergite in dorso-lateral view.

##### Body color.

Antenna brown; annulus absent; face, clypeus and gena yellow-orange; frons, temple and vertex dark brown. Propleuron dark brown except interior and posterior borders yellow; pronotum dorsally dark brown, ventrally yellow; mesonotal lobes black-dark brown, area between them and scutellum orange; mesopleuron dark brown close to the tegula, then gradually turns brown and light brown toward the middle coxa; metanotum dark brown; metapleuron light brown; propodeum dark brown. Prothoracic legs yellow; mesothoracic coxa, trochanter and trochantellus white, remaining leg dark brown; metathoracic coxa dorsally dark brown and ventrally yellow, trochanter, trochantellus and femur basally yellow, remaining leg brown. T1 black except the basal portion white-yellow; T2 basally yellow, remaining tergite surface brown; sterna yellow. Wings hyaline; stigma on front wing brown.

##### Body length.

4.4 mm.

##### Head.

Antenna with 31 flagellomeres; flagellar length/width ratios as follows: F1 = 3, F2 = 3, F3 = 2.6, F29 = 1.8, F30 = 1.5, F31 = 2; head 1.2 wider than high; occipital carina complete; ocellus-ocullar distance 1.1 × ocellar diameter; head height 1.5 × eye height; temple length 0.7 × eye length in dorsal view; vertex in dorsal view descending vertically behind the lateral ocelli; frons strigulate; face maximum width 1.1 × minimum width; face strigulate; face minimum width 1.2 × clypeus width; clypeus strigulate; malar space length 0.8 × mandible width basally; mandibles twisted.

##### Mesosoma.

Pronotum in lateral view rugose-foveate-carinate; propleuron mostly smooth except apically rugulose; notauli shallow, not distinctive and rugose with a pronounced longitudinal carina; mesonotal lobes well defined; central lobe of mesoscutum punctate; scutellar furrow with two carinae; mesopleuron mostly puncticulate, rugose close to the tegula; precoxal sulcus rugose-foveate; metapleuron mostly smooth, rugose close to the coxa; propodeum aerolate-carinate-rugose, longitudinal carina present, median depression absent.

##### Legs.

Hind coxa strigate; tarsal claw with large lobe.

##### Wings.

Wing length 4.2 mm; second submarginal cell of forewing not strongly narrowed anteriorly. Front wing: length of vein r 0.7 × length of vein 3RSa; vein 3RSb straight; length of vein 3RSa 0.9 × length of vein r-m; vein m-cu intersticial. Hind wing: length of vein 1M 0.9 × length of vein cu-a; length of vein 1M 0.7 × length of vein r-m.

##### Metasoma.

Dorsope absent; ventral borders of first tergite joined completely along ½ of segment; first tergite with costae parallel; ovipositor thickened basally and straight; ovipositor 2.3 × longer than first tergite.

##### Cocoon.

Unknown.

##### Female variation.

Unknown.

##### Male variation.

Unknown.

##### Type locality.

COSTA RICA, Heredia, Vara Blanca, Finca Georgina, 2100 m.

##### Type specimen.

Holotype female (point mounted), COSTA RICA, Heredia, Vara Blanca, Finca Georgina, 2100 m, collected III–IV.1990, Paul Hanson leg., UWIM.

Paratype. Unknown.

##### Distribution.

Costa Rica, province of Heredia.

##### Biology.

Unknown.

##### Comments.

*Meteorus martinezi* is similar to *Meteorus carolae* in having the occipital carina complete, mandibles totally twisted, notauli shallow and not distinct, tarsal claw with a large lobe, first metasomal tergite without dorsopes, ventral borders of first tergite joined along ½ of segment, mesopleuron completely brown-black, first tergite bicolored and propodeum totally black-dark brown. *Meteorus martinezi* can be separated from *Meteorus carolae* by the hind coxa dorsally dark brown and ventrally yellow (hind coxa completely dark brown in *Meteorus carolae*), antenna with 31 flagellomeres (antenna with 24–27 flagellomeres in *Meteorus carolae*) and the parallel eyes in frontal view, face maximum width/minimum width = 1.1 (convergent eyes in *Meteorus carolae*, face maximum width/minimum width = 1.4–1.6).

##### Etymology.

This species is named in honor of Dr. Juan Jose Martinez, Museo Argentino de Ciencias Naturales “Bernardino Rivadavia” curator of insects.

#### Meteorus microcavus

Taxon classificationAnimaliaHymenopteraBraconidae

Aguirre, Almeida & Shaw sp. n.

http://zoobank.org/7EDAF984-A3AC-42A4-97B5-18304638ABF3

Figures 128–134

##### Diagnosis.

Occipital carina complete; eyes convergent in frontal view, face maximum width 1.7 × minimum width; mandibles moderately twisted; notauli deeply impressed, distinctive and foveolate; propodeum carinate-rugose, with a transversal carina; hind coxa rugose; tarsal claw with a large lobe; dorsope present, very small; ventral borders of first tergite widely separated; ovipositor thickened basally and slightly curved; ovipositor 3.1 × longer than first tergite.

**Figures 128–134.**
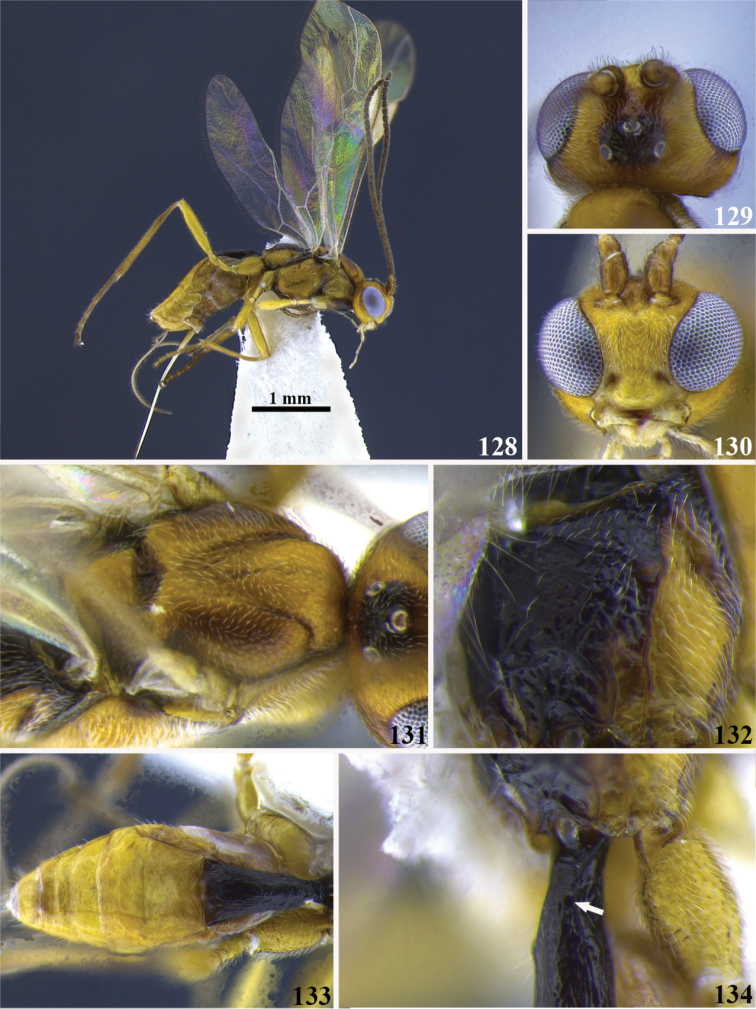
*Meteorus microcavus* sp. n. female. **128** Habitus in lateral view **129** head in dorsal view **130** head in frontal view **131** mesonotum in dorso-lateral view **132** propodeum in dorso-lateral view **133** metasoma in dorsal view **134** first tergite basal portion, the arrow shows the position of a small dorsope.

##### Body color.

Antenna brown; annulus absent; head yellow except area between ocelli dark brown. Anterior half of propleuron dark brown, posterior half light brown; pronotum yellow; median mesonotal lobe and scutellum yellow, lateral mesonotal lobes light brown; mesopleuron laterally yellow, ventrally light brown; metanotum black dorsally, yellow laterally; metapleuron yellow; propodeum black. Prothoracic legs yellow; mesothoracic coxa, trochanter and trochantellus white, remaining leg dark brown; metathoracic legs yellow except tarsus light brown. T1 black; T2–T8 and sterna yellow. Wings hyaline; stigma white.

##### Body length.

2.8 mm.

##### Head.

Antenna with 22 flagellomeres; head 1.2 wider than high; occipital carina complete; ocellus-ocullar distance 2 × ocellar diameter; head height 1.5 × eye height; temple length 0.5 × eye length in dorsal view; vertex in dorsal view not descending vertically behind the lateral ocelli; frons strigulate; face maximum width 1.7 × minimum width; face puncticulate; face minimum width 0.7 × clypeus width; clypeus smooth and polished; malar space length 0.5 × mandible width basally; mandibles moderately twisted.

##### Mesosoma.

Surface of pronotum in lateral view irregular and shiny; propleuron mostly smooth except anteriorly rugulose; notauli deeply impressed, distinctive and foveolate; mesonotal lobes well defined; central lobe of mesoscutum with irregular punctures and polished; scutellar furrow with one carina; mesopleuron with irregular punctures; precoxal sulcus short, narrow and foveate; metapleuron with irregular punctures; propodeum carinate-rugose, with a transversal carina.

##### Legs.

Hind coxa rugose; tarsal claw with a large lobe.

##### Wings.

Wing length 2.9 mm; second submarginal cell of forewing not strongly narrowed anteriorly. Front wing: length of vein r 0.9 × length of vein 3RSa; vein 3RSb straight; length of vein 3RSa 0.6 × length of vein r-m; vein m-cu antefurcal. Hind wing: length of vein 1M 1.6 × length of vein cu-a; length of vein 1M 1.2 × length of vein r-m.

##### Metasoma.

Dorsope present, very small; ventral borders of first tergite widely separated; first tergite costate-rugulose; ovipositor thickened basally and slightly curved; ovipositor 3.1 × longer than first tergite; T2–T3 with irregular and shiny surface.

##### Cocoon.

Unknown.

##### Female variation.

Unknown.

##### Male variation.

Unknown.

##### Type locality.

COSTA RICA, Cartago, Cerro de la Muerte, Villa Mills, 3000 m.

##### Type specimen.

Holotype female (point mounted), COSTA RICA, Cartago, Cerro de la Muerte, Villa Mills, 3000 m, collected XI–XII.1989, P. Hanson leg., UWIM.

Paratype. Unknown.

##### Distribution.

Costa Rica, province of Cartago.

##### Biology.

Unknown.

##### Comments.

Compared with *Meteorus fallacavus*, *Meteorus microcavus* displays a true pair of dorsopes but too small to be detected at a first glance. The ventral borders being widely separated support this interpretation. It is unusual to find such a reduction in these structures, so the conspicuous dorsopes diminution in *Meteorus microcavus* might be enough to identify it. *Meteorus andreae*, a common species distributed across the montane forests of Colombia and Costa Rica, matches with *Meteorus fallacavus* by sharing the following features: moderately twisted mandibles, propodeum having carinae, presence of true dorsopes, ventral borders of fist tergite widely separated. However, *Meteorus microcavus* differs by its mesopleuron mostly yellow (mesopleuron completely black in *Meteorus andreae*), antenna with 22 flagellomeres (antenna with 27–32 flagellomeres in *Meteorus andreae*) and tarsal claw with a large lobe (tarsal claw either simple or with a small lobe in *Meteorus andreae*).

##### Etymology.

The specific epithet is composed by the Greek prefix “micro” meaning small, and the Latin stem “cavus”, which means hole, referring to the small dorsopes.

#### Meteorus noctuivorus

Taxon classificationAnimaliaHymenopteraBraconidae

Aguirre, Almeida & Shaw sp. n.

http://zoobank.org/E03C841A-A1AD-4960-B7E4-2F8A8FA1906D

Figures 135–141 , 142–146

##### Diagnosis.

Occipital carina complete; big ocelli, ocellus-ocullar distance 0.8 × ocellar diameter; mandibles twisted; notauli shallow, not distinctive and rugose with a pronounced longitudinal carina; propodeum aerolate-rugose; dorsope absent; ventral borders of first tergite fused completely along ½ of segment; ovipositor 1.9 × longer than first tergite; mesopleuron completely yellow.

**Figures 135–141.**
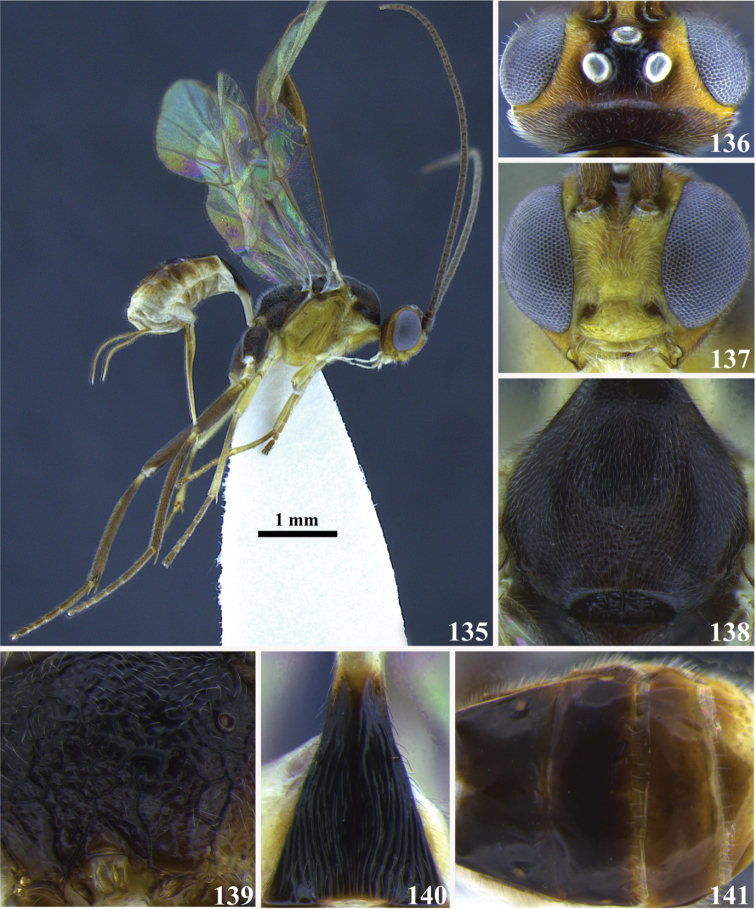
*Meteorus noctuivorus* sp. n. female. **135** Habitus in lateral view **136** head in dorsal view **137** head in frontal view **138** mesoscutum in dorsal view **139** propodeum in posterior view **140** first tergite in dorsal view **141** tergites T2–T5 in dorsal view.

**Figures 142–146.**
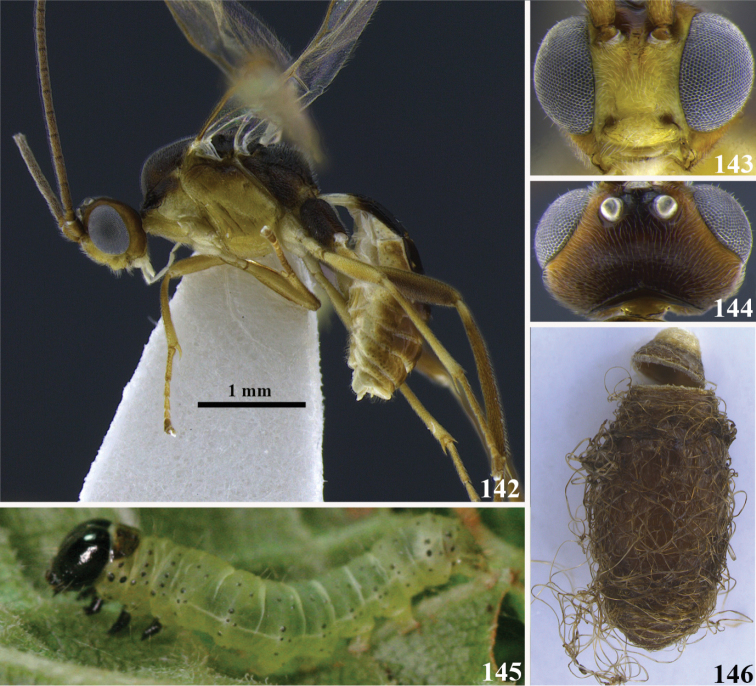
*Meteorus noctuivorus* sp. n. male. **142** Habitus in lateral view **143** head in frontal view **144** head in dorsal view **145** the *Meteorus noctuivorus*’ host, a noctuid caterpillar **146** cocoon.

##### Body color.

Antenna dark brown; annulus absent; head clypeus and face yellow; frons orange; gena orange infused with brown; vertex and occiput brown; area between ocelli black. Propleuron yellow; dorsal border of pronotum black, remaining yellow; mesonotum dark brown except scutellum testaceous; mesopleuron yellow; metanotum dark brown; metapleuron dark brown; propodeum black-dark brown. Prothoracic legs yellow except tarsus light brown; mesothoracic legs yellow except tibia apically and tarsus light brown; metathoracic legs brown except coxa dorsally dark brown and trochanter light brown. T1 white-yellow basally, dark brown apically; T2–T3 brown; T4–T5 light brown; T6–T8 yellow; sterna cream infused with light brown. Wings hyaline; stigma brown.

##### Body length.

4.5 mm.

##### Head.

Antenna with 29 flagellomeres; flagellar length/width ratios as follows: F1 = 4.2, F2 = 3.5, F3 = 3.3, F27 = 1.8, F28 = 2.2, F29 = 4.7; head 1.1 wider than high; occipital carina complete; ocellus-ocullar distance 0.8 × ocellar diameter; head height 1.5 × eye height; temple length 0.5 × eye length in dorsal view; vertex in dorsal view descending vertically behind the lateral ocelli; frons smooth and polished; face maximum width 1.2 × minimum width; face strigate-rugulose; face minimum width equal to clypeus width; clypeus rugulose-strigulate; malar space length 0.2 × mandible width basally; mandibles twisted.

##### Mesosoma.

Pronotum in lateral view carinate and rugose; propleuron irregular and shiny; notauli shallow, not distinctive and rugose with a pronounced longitudinal carina; mesonotal lobes not well defined; central lobe of mesoscutum punctuate; scutellar furrow with five carinae; mesopleuron puncticulate, rugose close to the tegula; precoxal sulcus short, narrow and rugose; metapleuron rugose; propodeum aerolate-rugose, neither carinae nor median depression present.

##### Legs.

Hind coxa strigate-rugulose; tarsal claw with large lobe.

##### Wings.

Wing length 4.4 mm; second submarginal cell of forewing not strongly narrowed anteriorly. Front wing: length of vein r 0.5 × length of vein 3RSa; vein 3RSb straight; length of vein 3RSa 0.9 × length of vein r-m; vein m-cu antefurcal. Hind wing: length of vein 1M 1.1 × length of vein cu-a; length of vein 1M 0.8 × length of vein r-m.

##### Metasoma.

Dorsope absent; ventral borders of first tergite fused completely along ½ of segment; first tergite basally smooth, apically with convergent costae; ovipositor thickened basally and straight; ovipositor 1.9 × longer than first tergite.

##### Cocoon.

Length cocoon 5.5 mm; width cocoon 2.4 mm; honey-brown translucent except apex cap golden, posteriorly bordered by a dark ring; oval-shaped, loosely wrapped by threads, end cap nipple-like, thread length 55 mm.

##### Female variation.

Unknown.

##### Male variation.

Mesonotum dark brown except a light brown patch posteriorly on scutellum; mesopleuron yellow except area close to the tegula dark brown; metapleuron brown except ventral borders light brown; prothoracic legs yellow; T2–T3 brown, remaining surface lighter; sterna yellow; head 1.2 wider than high; head height 1.4 × eye height; malar space length 0.4 × mandible width basally; propleuron disperse punctured; precoxal sulcus long, narrow and carinate-rugose; wing length 3.9 mm; length of vein r 0.9 × length of vein 3RSa; length of vein 3RSa 0.7 × length of vein r-m; length of vein 1M 1.1 × length of vein r-m.

##### Type locality.

ECUADOR, Napo province, Yanayacu biological station 00°35.9'S, 77°53.4'W, 2163 m.

##### Type specimen.

Holotype female (point mounted) ECUADOR, Napo province, Yanayacu biological station 00°35.9'S, 77°53.4'W, 2163 m, reared from a noctuid caterpillar collected on *Boehmeria bullata* (Urticaceae) IX.22.2010, parasitoid pupation X.13.2010, parasitoid emergence XI.3.2010, YY 51987 (rearing code), UWIM.

Paratype. Male, ECUADOR, Napo province, Yanayacu biological station, 00°35.9'S, 77°53.4'W, 2163 m, reared from a noctuid caterpillar collected on *Boehmeria bullata* (Urticaceae) IX.5.2010, parasitoid pupation IX.29.2010, parasitoid emergence X.26.2010, YY 51587 (rearing code), UWIM.

##### Distribution.

Ecuador, province of Napo.

##### Biology.

Solitary parasitoid of a noctuid caterpillar feeding on *Boehmeria bullata* (Urticaceae)

##### Comments.

*Meteorus noctuivorus* and *Meteorus anuae* share the occipital carina being complete, mandibles completely twisted, notauli shallow and not distinct, tarsal claw with a large lobe, ventral borders of first tergite joined along half of segment and first metasomal tergite without dorsopes. *Meteorus noctuivorus* might be distinguished by the first tergite basally white-yellow, distally brown-black (first tergite completely black in *Meteorus anuae*).

##### Etymology.

The stem “noctui” (referring to the host family) and the suffix “vorus” meaning devouring, compose the specific epithet (“the noctuid-devourer”).

#### Meteorus orion

Taxon classificationAnimaliaHymenopteraBraconidae

Aguirre, Almeida & Shaw sp. n.

http://zoobank.org/689D3A0B-1980-40C4-9A0A-857105D30DDF

Figures 147–153

##### Diagnosis.

Occipital carina incomplete; mandibles twisted; notauli rugose-carinate and not distinct; longitudinal and transversal carinae on propodeum forming broad areolae dorsally; hind coxa strigate and punctate; tarsal claw simple; dorsope absent; ventral borders of first tergite joined completely along ½ of segment; ovipositor 1.7 × longer than first tergite; colorful pattern of orange, yellow, white and black on the body.

**Figures 147–153.**
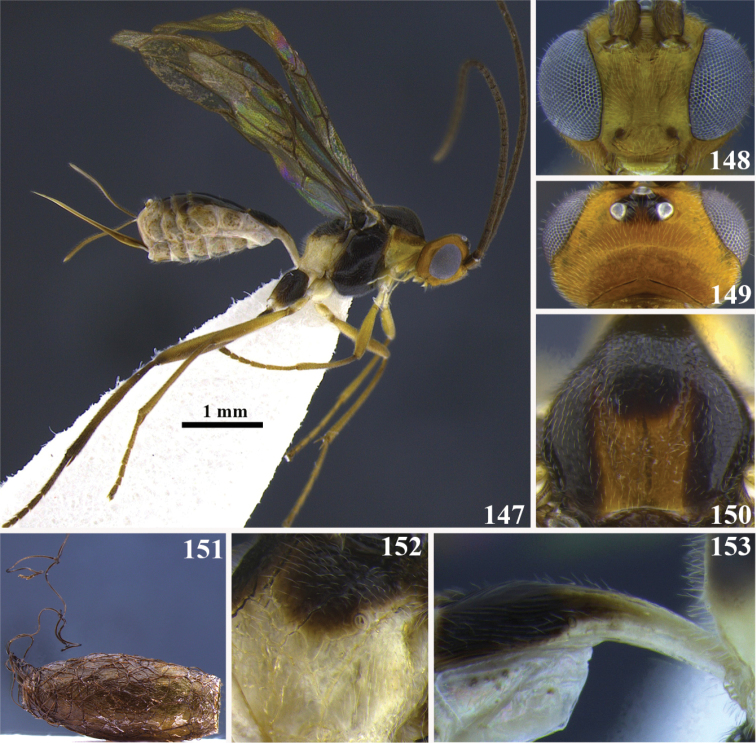
*Meteorus orion* sp. n. female. **147** Habitus in lateral view **148** head in frontal view **149** head in dorsal view **150** mesoscutum in dorsal view **151** cocoon **152** propodeum in dorso-lateral view **153** first tergite in dorso-lateral view.

##### Body color.

Antenna dark brown; annulus absent; head orange except area between ocelli black. Propleuron orange-yellow; pronotum dorsally orange, ventrally yellow; mesonotum dark brown, except area among lobes and a patch on scutellum orange; mesopleuron dark brown; metanotum dark brown; metapleuron white; propodeum dark brown except posterior and lateral areas white-cream. Prothoracic legs testaceous except coxa and trochanter white cream; mesothoracic legs testaceous except coxa and trochanter white cream; metathoracic legs dark brown except entire femur and tibia medially testaceous. T1 white-yellow basally, dark brown apically; T2–T8 dark brown; sterna yellow-cream with dark brown spots. Wings hyaline; stigma brown.

##### Body length.

3.9 mm.

##### Head.

Antenna with 29 flagellomeres; flagellar length/width ratios as follows: F1 = 3.4, F2 = 3.1, F3 = 3.1, F27 = 1.8, F28 = 1.7, F29 = 2.2; head 1.3 wider than high; occipital carina incomplete; ocellus-ocullar distance 1.6 × ocellar diameter; head height 1.6 × eye height; temple length 0.4 × eye length in dorsal view; vertex in dorsal view not descending vertically behind the lateral ocelli; frons smooth and polished; face maximum width 1.2 × minimum width; face strigate-punctate; face minimum width 1.3 × clypeus width; clypeus rugose; malar space length 1.1 × mandible width basally; mandibles twisted.

##### Mesosoma.

Pronotum in lateral view carinate-punctate; propleuron slightly puncticulate; notauli rugose-carinate and not distinct; mesonotal lobes not well defined. central lobe of mesoscutum rugulose; scutellar furrow with three carinae; mesopleuron punctate, rugose-lacunose close to the tegula; precoxal sulcus long, wide and carinate-rugose; metapleuron rugulose; propodeum carinate-rugose; longitudinal and transversal carinae forming broad areolae dorsally, median depression absent.

##### Legs.

Hind coxa strigate and punctate; tarsal claw simple.

##### Wings.

Wing length 3.4 mm; second submarginal cell of forewing not strongly narrowed anteriorly. Front wing: length of vein r 0.7 × length of vein 3RSa; vein 3RSb straight; length of vein 3RSa 0.9 × length of vein r-m; vein m-cu postfurcal. Hind wing: length of vein 1M equal to length of vein cu-a; length of vein 1M 1.4 × length of vein r-m.

##### Metasoma.

Dorsope absent; ventral borders of first tergite joined completely along ½ of segment; first tergite with costae convergent posteriorly; ovipositor thickened basally and straight; ovipositor 1.7 × longer than first tergite.

##### Cocoon.

Length cocoon 3.9 mm; width cocoon 1.8 mm; honey-brown translucent. Oval-shaped, main structure formed by honey-light brown threads, loosely enveloped by darker threads.

##### Female variation.

Unknown.

##### Male variation.

Unknown.

##### Type locality.

ECUADOR, Napo province, Yanayacu biological station, San Isidro forest, 00°35.9'S; 77°53.4'W, 2163 m.

##### Type specimen.

Holotype female (point mounted), ECUADOR, Napo province, Yanayacu biological station, San Isidro forest, 00°35.9'S; 77°53.4'W, 2163 m, reared from a noctuid caterpillar collected on Diplazium costale var robustum (Dryopteridaceae) VII.17.2009, parasitoid pupation VII.21.2009, parasitoid emergence VIII.7.2009, YY40067 (rearing code), UWIM.

Paratype. Unknown.

##### Distribution.

Ecuador, province of Napo.

##### Biology.

Solitaty parasitoid of Noctuidae feeding on Diplazium costale var. robustum (Dryopteridaceae).

##### Comments.

The occipital carina incomplete, mandibles completely twisted, first metasomal tergite without dorsopes, ventral borders of first tergite joined along half of segment and the colorful pattern of orange, yellow, black and white on the body set *Meteorus orion* close to *Meteorus mirandae*. The new species might be easily sorted by having the hind coxa completely dark brown and the middle one completely yellowish-white (hind and middle coxae dorsally black, ventrally yellow in *Meteorus mirandae*), the notauli shallow and not distinct, and the tarsal claw simple.

##### Etymology.

The mythological Greek hunter “Orion” inspired the name for this species, because of the hunting behavior upon noctuid caterpillars. By coincidence, the yellowish white middle coxa line up with the pale white posterior of the propodeum, like the three stars in the “belt of Orion,” the most conspicuous part of this famous constellation.

### New distribution and biology records

#### Meteorus andreae

Taxon classificationAnimaliaHymenopteraBraconidae

Aguirre & Shaw, 2011

##### Material examined.

One female (point mounted), COSTA RICA, Guanacaste, Volcán Cacao, Cerro Pedregal, 1000 m, collected II–IV.1989, I. Gauld and D. Janzen leg., UWIM. One female (point mounted), COSTA RICA, San José, Cerro de la Muerte, 26 km N San Isidro, 2100 m, collected II–V.1991, P. Hanson leg., UWIM. One female (point mounted), COSTA RICA, Puntarenas, San Vito, Estac. Biol. Las Alturas, 1500 m, collected XII.1991, P. Hanson leg., UWIM. One female (point mounted), COSTA RICA, Cartago, La Cangreja, 1950 m, collected VII.1991, P. Hanson leg., Malaise, UWIM. One female (point mounted), COSTA RICA, San José, Cerro de la Muerte, 2100 m, collected II–V.1992, P Hanson leg., Malaise, UWIM. One female (point mounted), COSTA RICA, Cartago, Cerro de la Muerte, 3000 m, collected XII.1988–I.1989, P. Hanson leg., Malaise, UWIM. One male (point mounted), COSTA RICA, San José, San Isidro, 2100 m, collected II–IV.1993, P. Hanson leg., Malaise, UWIM. One female (point mounted), COSTA RICA, Alajuela, San Ramón, 1200 m, collected collected II.1997, P. Hanson leg., Malaise, UWIM. One male (point mounted), COSTA RICA, Alajuela, San Ramón, 1200 m, collected VII.1997, P. Hanson leg., Malaise, UWIM.

##### Comments.

*Meteorus andreae* is one of the most common species of *Meteorus* in Costa Rica with approximately 200 specimens collected across five out of seven provinces, ranging from 745–3000 m above the sea level. It was originally described from Colombia in the departments of Cauca, Huila and Nariño, spanning between 1885–2640 m (Aguirre et al. 2011).

#### Meteorus farallonensis

Taxon classificationAnimaliaHymenopteraBraconidae

Aguirre & Shaw, 2011

##### Material examined.

Two females (point mounted), COSTA RICA, Puntarenas, Zona protectora Las tablas, 1 km NE de Sitio Portones Camino a Tablas, 1530 m, collected 30.VIII–5.IX.1995, M. Chinchilla, Malaise, UWIM. One female (point mounted), COSTA RICA, Puntarenas, San Vito, Est. Biol. Las Alturas, 1500 m, collected II.1992, P. Hanson leg., UWIM.

##### Comments.

*Meteorus farallonensis* was described from Colombia from the departments of Caqueta, Meta, and Valle del Cauca at elevations below 1000 m (Aguirre et al. 2011). This new record from Puntarenas, Costa Rica, at 1500 m represents the highest known altitudinal distribution for this species.

#### Meteorus guineverae

Taxon classificationAnimaliaHymenopteraBraconidae

Aguirre & Shaw, 2011

##### Material examined.

One female (point mounted), COSTA RICA, Cartago, La Cangreja, 1950 m, collected XI.1991, P. Hanson leg., UWIM. One female (point mounted), COSTA RICA, Heredia, Vara Blanca, Finca Georgina, 2100 m, collected I–II.1990, P. Hanson leg., UWIM. One female (point mounted), COSTA RICA, San José, Zurqui de Moravia, 1600 m, collected II.1996, P. Hanson leg., Malaise, UWIM.

##### Comments.

The type series was described from the Fauna and Flora Sanctuary of Iguaque, a high Andean fog forest, 2855–3350 m (Aguirre et al. 2011). This is the first record from outside Colombia.

#### Meteorus jerodi

Taxon classificationAnimaliaHymenopteraBraconidae

Aguirre & Shaw, 2011

##### Material examined.

Seventeen females, one male (point mounted), ECUADOR, Province of Napo 00°43'52.5"S, 77°46'25.3"W, Narupa, 1186 m, collected as a noctuid caterpillar parasitoid feeding on Asteraceae 3.IV.2013, pupated 15.IV.2013, emerged 29.V.2013, YY73611 (rearing code), UWIM.

##### Comments.

This species is known from the locality of Zipacón (1425 m), department of Cundinamarca, and from the locality of Togii (1830 m), department of Boyacá, Colombia (Aguirre et al. 2011). *Meteorus jerodi* was described from Malaise traps samples and the information here provided represents its first biological record.

#### Meteorus kraussi

Taxon classificationAnimaliaHymenopteraBraconidae

Muesebeck, 1958

##### Material examined.

One female (point mounted), COSTA RICA, San Jose, Zurqui de Moravia, 1600m, collected VIII.1995, P. Hanson leg., UWIM. One female pin mounted, COSTA RICA, Guanacaste, Est. Pitilla, 9 km S de Santa Cecilia, 700 m, collected VIII–IX.1996, P. Rios and C. Moraga leg., UWIM. One female (point mounted), COSTA RICA, Puntarenas, San Vito, Est. Biol. Las Alturas, 1500 m, collected VI.1992, P. Hanson leg., UWIM. One female pin mounted, COSTA RICA, Alajuela, 5 km W San Ramón, 1200 m, collected IV.1997, O. Castro and P. Hanson leg., UWIM.

##### Comments.

The type series was described from Cuernavaca, Mexico, 23 females and 3 males reared from a lepidopterous larva on *Ageratina adenophora* (Spreng.) King & H.Rob. (syn. *Eupatorium adenophorum*) (Muesebeck 1958). This is the first record outside Mexico since its original description.

#### Meteorus papiliovorus

Taxon classificationAnimaliaHymenopteraBraconidae

Zitani, 1997

##### Material revised.

Seventy one females (point mounted), ECUADOR, Napo, 00°43'52.5"S, 77°46'25.3"W, Narupa, sendero Alucus, 1186 m, each wasp was collected as a solitary parasitoid on individual larvae of Papilionidae “popo de pajaro” 14.IX.2013 feeding on a lemon tree *Citrus* sp. (Rutaceae); all parasitoids larvae pupated 2.X.2013; 11 wasps emerged 24.IX.2013, one emerged 27.IX.2013, five emerged 30.IX.2013, two emerged 1.X.2013, 39 emerged 7.X.2013, three emerged 8.X.2013, five emerged 9.X.2013, two emerged 10.X.2013 and three emerged 14.X.2013; rearing codes: YY 80190–202, 80204–209, 80211–217, 80222, 80224, 80226–229, 80231–233, 80235–236, 80238–244, 80246–247, 80249–251, 80254, 80257, 80261–268, 80271–275, 80277–282, 80284, UWIM.

##### Comments.

*Meteorus papiliovorus* Zitani represents the first Neotropical member of this genus known to have a strong preference for Papilionidae: originally described from Costa Rica parasitizing *Parides sesostris zestos* (Gray) and *Papilio anchisiades idaeus* (Fabricius, 1793) in 1997 (Zitani et al. 1997), and reared in 1946 in Colombia parasitizing *Papilio anchisiades capis* (Hübner) and in 1999 parasitizing *Papilio anchisiades idaeus* (Aguirre et al. 2011).

#### Meteorus quimbayensis

Taxon classificationAnimaliaHymenopteraBraconidae

Aguirre & Shaw, 2011

##### Material revised.

One female (point mounted), ECUADOR, Napo, 00°35.9'S, 77°53.4'W, Yanayacu Biological Station, J. Simbaña Macucoloma trail, 2163 m, collected 1–10.V.2009, S.R. Shaw leg., Malaise, UWIM. One female (point mounted), ECUADOR, Napo, 00°35.9'S, 77°53.4'W, Yanayacu Biological Station, J. Simbaña Macucoloma trail, 2163 m, collected 1–8.IX.2007, S.R. Shaw leg., Malaise, UWIM.

##### Comments.

*Meteorus quimbayensis*, originally described from Colombia from the departments of Huila, Risaralda, and Santander, it seems to be restricted to high South American Andean wet forests between 2000–2300 m above the sea level (Aguirre et al. 2011) since it has not been recorded from Costa Rica despite the intense sampling effort in locations such as Cerro de la Muerte reaching between 2100–3000 m.

## Host use in *Meteorus*

**Figure 154.**
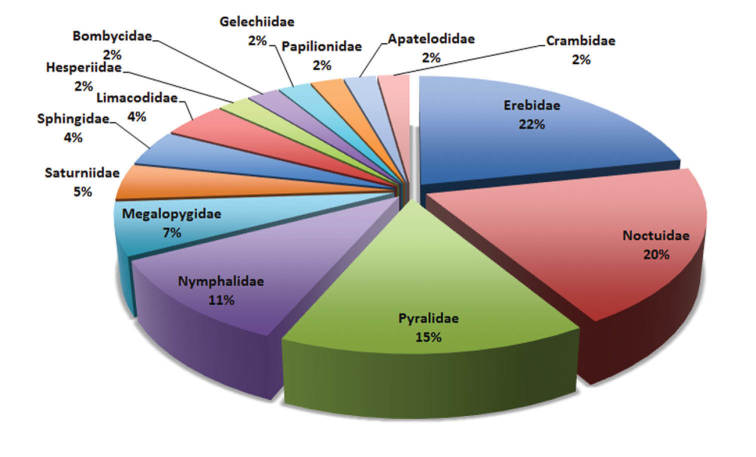
Percentages of host families known to be parastized by *Meteorus* species in Neotropical countries.

Biological information for 38 out of 75 *Meteorus* species is available (Table 1). Erebidae, Noctuidae and Pyralidae account for 57% of host records (Fig. 154). The highest percentage is kept by the familily Erebidae (22%) reported mainly from Ecuador as a result of the CAPEA project (Dyer et al. 2014). By contrast, Noctuidae with 20% of host records is reported from eight countries, from Mexico to Argentina, chiefly because of the tight association of noctuid caterpillars with commercial crops (Molina-Ochoa et al. 2003). Nineteen species are recorded as developing gregariously, sixteen as solitary and two present both behaviors. Gregarious *Meteorus* seem to display some preference toward caterpillars with physical and chemical defenses dissuading predators since six out of ten species (60%) attacking tiger moths larvae are gregarious compared to three out of nine (33.3%) parasitizing Noctuidae, one out of seven (14,3%) attacking Pyralidae, and one out of five (20%) species doing it on Nymphalidae. The most common and widespread species, *Meteorus laphygmae* Viereck, is also the most generalist species, using Erebidae, Nymphalidae, and Noctuidae as hosts.

**Table 1.** Distribution, host records and larvae development of Neotropical *Meteorus*. The last column provides information about the examined material and its repository. The superscripts indicate the following references: ^1^Aguirre and Shaw 2014a, ^2^Aguirre et al. 2011, ^3^Zitani et al. 1998, ^4^Aguirre and Shaw 2014b, ^5^Jones and Shaw 2012, ^6^Cave 1993, ^7^Maes 1989, ^8^Hilburn et al. 1990, ^9^Pair et al. 1986, ^10^Porter 1926, ^11^De Huiza 1994, ^12^De Santis 1967, ^13^Artigas 1972, ^14^Muesebeck 1939, ^15^Muesebeck 1958, ^16^Aguirre et al. 2010, *Molina-Ochoa et al. 2003 erroneous record, misinterpretation of Etcheverry 1957, ^17^Marsh 1979, ^18^Ortegón et al. 1988, ^19^Gladstone 1991, ^20^Dyer et al. 2005, ^21^Segeren and Sharma 1978, ^22^Muesebeck 1967, ^23^Muesebeck 1923, ^24^Shaw and Nishida 2005, ^25^Barrantes et al. 2011, ^26^Luna and Sanchez 1999, ^27^Shaw and Jones 2009, ^28^Molina-Ochoa et al. 2001, ^29^Ashmead 1889. Both distribution and host information without superscript are new records.

Parasitoid species	Distribution	Host family	Mode of parasitoid development	Material examined (Depository)
*Meteorus albisericus*	Ecuador^1^	Pyralidae^1^	Solitary^1^	Holotype (UWIM)
*Meteorus albistigma* sp. n.	Costa Rica	Unknown	Unknown	Holotype (UWIM)
*Meteorus alejandromasisi*	Colombia^2^, Costa Rica^3^	Hesperiidae^3^, Megalopygidae^2^	Gregarious^3^	Holotype (UWIM)
*Meteorus amazonensis*	Colombia^2^	Unknown	Unknown	Holotype (ICN)
*Meteorus andreae*	Colombia^2^, Costa Rica	Unknown	Unknown	Holotype (ICN)
*Meteorus antioquensis*	Colombia^2^	Saturniidae^2^	Gregarious^2^	Paratype (ICN)
*Meteorus anuae*	Ecuador^4^	Erebidae^4^	Gregarious^4^	Holotype (UWIM)
*Meteorus arizonensis*	Colombia^2^, Costa Rica, Honduras^6^, Nicaragua^7^	Noctuidae^6,7^	Unknown	Voucher (UWIM)
*Meteorus autographae*	Bermuda^8^, Mexico^9^	Noctuidae^9^	Solitary^23^	Voucher (UWIM)
*Meteorus boyacensis*	Colombia^2^	Unknown	Unknown	Holotype (ICN)
*Meteorus bustamanteorum*	Ecuador^5^	Bombycidae^5^	Gregarious^5^	Holotype (UWIM)
*Meteorus calimai*	Colombia^2^	Unknown	Unknown	Holotype (ICN)
*Meteorus camilocamargoi*	Costa Rica^3^	Pyralidae^3^	Solitary^3^	Holotype (UWIM)
*Meteorus caquetensis*	Colombia^2^	Unknown	Unknown	Holotype (ICN)
*Meteorus caritatis*	Ecuador^5^	Nymphalidae^5^	Solitary^5^	Holotype (UWIM)
*Meteorus carolae* sp. n.	Costa Rica	Unknown	Unknown	Holotype (UWIM)
*Meteorus cecavorum*	Colombia^2^, Ecuador^4^	Erebidae^4^	Gregarious^4^	Holotype (ICN)
*Meteorus chilensis*	Argentina^12^, Chile^10,13^, Peru^11^	Noctuidae^11,13^	Gregarious^11^	Voucher (UWIM)
*Meteorus chingazensis*	Colombia^2^	Unknown	Unknown	Holotype (ICN)
*Meteorus coffeatus*	Costa Rica^3^	Unknown	Unknown	Holotype (UWIM)
*Meteorus congregatus*	Costa Rica^3^, Panama^14^	Sphingidae^14^	Gregarious^14^	Paratype (NMNH)
*Meteorus corniculatus*	Colombia^2^, Costa Rica^3^	Unknown	Unknown	Holotype (UWIM)
*Meteorus desmiae*	Colombia^2^, Costa Rica^3^, Ecuador^1^	Pyralidae^1^, Crambidae^1^	Solitary^3^	Holotype (UWIM)
*Meteorus dimidiatus*	Colombia^2^, Costa Rica^3^	Unknown	Unknown	Voucher (UWIM)
*Meteorus dixi*	Colombia^2^	Unknown	Unknown	Holotype (ICN)
*Meteorus dos*	Colombia^2^, Costa Rica^3^	Unknown	Unknown	Holotype (UWIM)
*Meteorus eaclidis*	Brazil^15^	Saturniidae^15^	Gregarious^15^	Paratype (NMNH)
*Meteorus euchromiae*	Venezuela^29^	Erebidae^29^	Unknown	Paratype (NMNH)
*Meteorus eurysaccavorus* sp. n.	Bolivia	Gelechiidae	Unknown	Holotype (UWIM)
*Meteorus fallacavus* sp. n.	Costa Rica	Unknown	Unknown	Holotype (UWIM)
*Meteorus farallonensis*	Colombia^2^, Costa Rica	Unknown	Unknown	Holotype (ICN)
*Meteorus flavistigma* sp. n.	Costa Rica	Unknown	Unknown	Holotype (UWIM)
*Meteorus gigas*	Colombia^16^, Ecuador^16^	Unknown	Unknown	Paratype (UWIM)
*Meteorus guacharensis*	Colombia^2^	Unknown	Unknown	Holotype (ICN)
*Meteorus guineverae*	Colombia^2^, Costa Rica	Unknown	Unknown	Holotype (ICN)
*Meteorus haimowitzi* sp. n.	Costa Rica	Unknown (reared from cocoon)	Solitary	Holotype (UWIM)
*Meteorus horologium*	Ecuador^5^	Limacodidae^5^	Gregarious^5^	Holotype (UWIM)
*Meteorus huilensis*	Colombia^2^	Unknown	Unknown	Holotype (ICN)
*Meteorus iguaquensis*	Colombia^2^	Unknown	Unknown	Holotype (ICN)
*Meteorus imaginatus*	Ecuador^5^	Noctuidae^5^	Solitary^5^	Holotype (UWIM)
*Meteorus jerodi*	Colombia^2^, Ecuador	Noctuidae	Gregarious	Holotype (ICN)
*Meteorus juliae*	Ecuador^4^	Erebidae^4^	Gregarious^4^	Holotype (UWIM)
*Meteorus kraussi*	Mexico^15^, Costa Rica	Unknown	Gregarious^15^	Paratype (NMNH)
*Meteorus laphygmae*	Chile*, Colombia^18^, Costa Rica^3^, Honduras^6^, Mexico^17,28^, Nicaragua^19^, Suriname^21^, Venezuela^22^	Nymphalidae^20^, Noctuidae^6,17,18,19^, Erebidae^20^	Solitary^23^	Voucher (UWIM)
*Meteorus luteus*	Ecuador^5^	Nymphalidae^5^	Solitary^5^	Holotype (UWIM)
*Meteorus magdalensis*	Colombia^2^	Unknown	Unknown	Holotype (ICN)
*Meteorus magnoculus* sp. n.	Costa Rica	Pyralidae	Unknown	Holotype (UWIM)
*Meteorus margarita*	Ecuador^5^	Erebidae^5^	Gregarious^5^	Holotype (UWIM)
*Meteorus mariamartae*	Colombia^2^, Costa Rica^3^	Unknown	Unknown	Holotype (UWIM)
*Meteorus martinezi* sp. n.	Costa Rica	Unknown	Unknown	Holotype (UWIM)
*Meteorus megalops*	Colombia^2^, Costa Rica^3^	Unknown	Unknown	Holotype (UWIM)
*Meteorus microcavus* sp. n.	Costa Rica	Unknown	Unknown	Holotype (UWIM)
*Meteorus micrommatus*	Costa Rica^3^	Unknown	Unknown	Holotype (UWIM)
*Meteorus mirandae*	Ecuador^4^	Erebidae^4^	Solitary^4^	Holotype (UWIM)
*Meteorus muiscai*	Colombia^2^	Unknown	Unknown	Holotype (ICN)
*Meteorus noctuivorus* sp. n.	Ecuador	Noctuidae	Solitary	Holotype (UWIM)
*Meteorus oreo*	Ecuador^5^	Erebidae^5^	Solitary^5^	Holotype (UWIM)
*Meteorus orion* sp. n.	Ecuador	Noctuidae	Solitary	Holotype (UWIM)
*Meteorus oviedoi*	Colombia^2^, Costa Rica^24^	Limacodidae^24^	Gregarious^24^	Holotype (UWIM)
*Meteorus papiliovorus*	Colombia^2^, Costa Rica^25^, Ecuador	Papilionidae^2,25^, Nymphalidae^2^	Gregarious^2,25^ Solitary	Holotype (UWIM)
*Meteorus porcatus*	Ecuador^5^	Erebidae^5^	Gregarious^5^	Holotype (UWIM)
*Meteorus pseudodimidiatus*	Colombia^2^, Costa Rica^3^	Unknown	Unknown	Holotype (UWIM)
*Meteorus pyralivorus*	Ecuador^1^	Pyralidae^1^	Solitary^1^	Holotype (UWIM)
*Meteorus quasifabatus*	Ecuador^5^	Erebidae^5^	Gregarious^5^	Holotype (UWIM)
*Meteorus quimbayensis*	Colombia^2^, Ecuador	Unknown	Unknown	Holotype (ICN)
*Meteorus restionis*	Costa Rica^25^	Unknown (reared from cocoon)	Gregarious^25^	Holotype (UWIM)
*Meteorus rogerblancoi*	Colombia^2^, Costa Rica^3^	Unknown	Unknown	Holotype (UWIM)
*Meteorus rubens*	Argentina^26^, Colombia^2^, Costa Rica^3^	Megalopygidae^3^, Noctuidae^2,26^, Pyralidae^26^	Solitary^26^, Gregarious^3^	Voucher (UWIM)
*Meteorus rugonasus*	Colombia^2^, Ecuador^27^	Nymphalidae^27^	Solitary^27^	Holotype (UWIM)
*Meteorus santanderensis*	Colombia^2^	Unknown	Unknown	Holotype (ICN)
*Meteorus sterictae*	Costa Rica^3^	Pyralidae^3^	Solitary^3^	Holotype (UWIM)
*Meteorus townsendi*	Brazil^14^, Colombia^2^	Sphingidae^14^	Gregarious^14^	Paratype (NMNH)
*Meteorus uno*	Colombia^2^, Costa Rica^3^	Unknown	Unknown	Holotype (UWIM)
*Meteorus yamijuanum*	Colombia^2^, Costa Rica^3^	Unknown	Unknown	Holotype (UWIM)
*Meteorus zitaniae*	Ecuador^5^	Megalopygidae^5^	Gregarious^5^	Holotype (UWIM)

## Supplementary Material

XML Treatment for Meteorus albistigma

XML Treatment for Meteorus carolae

XML Treatment for Meteorus eurysaccavorus

XML Treatment for Meteorus fallacavus

XML Treatment for Meteorus flavistigma

XML Treatment for Meteorus haimowitzi

XML Treatment for Meteorus magnoculus

XML Treatment for Meteorus martinezi

XML Treatment for Meteorus microcavus

XML Treatment for Meteorus noctuivorus

XML Treatment for Meteorus orion

XML Treatment for Meteorus andreae

XML Treatment for Meteorus farallonensis

XML Treatment for Meteorus guineverae

XML Treatment for Meteorus jerodi

XML Treatment for Meteorus kraussi

XML Treatment for Meteorus papiliovorus

XML Treatment for Meteorus quimbayensis

## Supplementary materials

Supplementary material 1 Revised material Data type: Excel spreadsheet. Explanation note: Data set containing information about parasitoid species, host species, distribution, trapping method and depository. This dataset is made available under the Open Database License (http://opendatacommons.org/licenses/odbl/1.0/). The Open Database License (ODbL) is a license agreement intended to allow users to freely share, modify, and use this Dataset while maintaining this same freedom for others, provided that the original source and author(s) are credited.Helmuth Aguirre, Luis Felipe de Almeida, Scott Richard Shaw, Carlos E. Sarmiento
